# A Small Molecule, Which Competes with MAdCAM-1, Activates Integrin α_4_β_7_ and Fails to Prevent Mucosal Transmission of SHIV-SF162P3

**DOI:** 10.1371/journal.ppat.1005720

**Published:** 2016-06-27

**Authors:** Géraldine Arrode-Brusés, Diana Goode, Kyle Kleinbeck, Jolanta Wilk, Ines Frank, Siddappa Byrareddy, James Arthos, Brooke Grasperge, James Blanchard, Thomas Zydowsky, Agegnehu Gettie, Elena Martinelli

**Affiliations:** 1 Center for Biomedical Research, Population Council, New York, New York, United States of America; 2 University of Nebraska Medical Center, Omaha, Nebraska, United States of America; 3 Laboratory of Immunoregulation, National Institute of Allergy and Infectious Diseases, Bethesda, Maryland, United States of America; 4 Tulane National Primate Research Center, Tulane University, Covington, Louisiana, United States of America; 5 Aaron Diamond AIDS Research Center, Rockefeller University, New York, New York, United States of America; Emory University, UNITED STATES

## Abstract

Mucosal HIV-1 transmission is inefficient. However, certain viral and host characteristics may play a role in facilitating HIV acquisition and systemic expansion. Cells expressing high levels of integrin α_4_β_7_ have been implicated in favoring the transmission process and the infusion of an anti-α_4_β_7_ mAb (RM-Act-1) prior to, and during a repeated low-dose vaginal challenge (RLDC) regimen with SIVmac251 reduced SIV acquisition and protected the gut-associated lymphoid tissues (GALT) in the macaques that acquired SIV. α_4_β_7_ expression is required for lymphocyte trafficking to the gut lamina propria and gut inductive sites. Several therapeutic strategies that target α_4_β_7_ have been shown to be effective in treating inflammatory conditions of the intestine, such as inflammatory bowel disease (IBD). To determine if blocking α_4_β_7_ with ELN, an orally available anti-α_4_ small molecule, would inhibit SHIV-SF162P3 acquisition, we tested its ability to block MAdCAM-1 (α_4_β_7_ natural ligand) and HIV-gp120 binding in vitro. We studied the pharmacokinetic profile of ELN after oral and vaginal delivery in macaques. Twenty-six macaques were divided into 3 groups: 9 animals were treated with ELN orally, 9 orally and vaginally and 8 were used as controls. All animals were challenged intra-vaginally with SHIV-SF162P3 using the RLDC regimen. We found that ELN did not protect macaques from SHIV acquisition although it reduced the SHIV-induced inflammatory status during the acute phase of infection. Notably, integrins can exist in different activation states and, comparing the effect of ELN and the anti-α_4_β_7_ mAb RM-Act-1 that reduced susceptibility to SIV infection, we determined that ELN induces the active conformation of α_4_β_7_, while RM-Act-1 inhibits its activation through an allosteric mechanism. These results suggest that inhibition of α_4_β_7_ activation may be necessary to reduce susceptibility to SIV/SHIV infection and highlight the complexity of anti-integrins therapeutic approach in HIV as well as in IBD and other autoimmune diseases.

## Author Summary

To successfully infect a new host through the sexual route, HIV needs to travel to anatomical sites distant from the mucosal site of exposure reaching draining lymph nodes and the gut, where it can expand and disseminate. The characteristics of the vaginal mucosal microenvironment that facilitate HIV acquisition are still unclear. Several lines of evidence suggest that the ability of HIV to infect cells expressing integrin α_4_β_7_, a receptor that normally guides immune cells to the gut, may constitute an advantage during transmission and blocking α_4_β_7_ with a laboratory-engineered antibody (mAb) was shown to reduce susceptibility to vaginal SIV infection. However, α_4_β_7_ can exist in different conformational states that can affect cell function and susceptibility to infection. Herein we show that while the anti-α_4_β_7_ mAb that reduced susceptibility to infection inhibits α_4_β_7_ activation, a drug that also binds to α_4_β_7_, but induces its activation does not decrease susceptibility to SHIV infection. Thus, our results suggest that not only α_4_β_7_ expression, but also its activation state may play a role in facilitating or inhibiting infection. Our study contributes to the understanding of mechanisms that facilitate HIV transmission, suggesting innovative ways to prevent it.

## Introduction

HIV mucosal transmission requires the expansion of a small population of infected cells that have to reach draining lymph nodes (LNs) and the gut associated lymphoid tissues (GALT) to support viral amplification and systemic dissemination. Leukocyte migration to the gut tissue and the GALT is mediated primarily by integrin α_4_β_7_, an heterodimeric receptor that binds to mucosal vascular addressin cell adhesion molecule-1 (MadCAM-1) on high endothelial venules (HEVs) of Peyers patches (PPs) and mesenteric lymph nodes (MLNs) as well as on postcapillary venules of gut lamina propria (LP) [1, 2]. In the multistep model of leukocyte binding to endothelium and migration into tissues, it is generally selectins that mediate tethering and rolling on the vessel wall and integrins that mediate subsequent firm adhesion and migration [3, 4]. The largest exception to this rule is integrin α_4_β_7_, which mediates both rolling and firm adhesion in vivo as it functions as a gut homing receptor [5]. Several lines of evidence suggest that CD4^+^ T cells expressing high levels of α_4_β_7_ (α_4_β_7_^high^) play a critical role in HIV/SIV infection. They are the preferential targets of HIV/SIV infection and increased frequencies of α_4_β_7_^high^ CD4^+^ T cells at the time of challenge appear to correlate with increased susceptibility to rectal SIV infection and increased plasma viral loads (VLs) [6–11]. Moreover, prevalent HSV-2 infection and high progesterone levels, which are associated with higher risk of HIV-1 acquisition [12, 13], increase the frequency of α_4_β_7_^high^ CD4^+^ T cells in the female genital tract and rectal tissue [9, 14, 15]. A specific interaction between HIV and SIV gp120s and α_4_β_7_ has been described. However, the relevance of this interaction in HIV transmission and pathogenesis is still debated [16, 17]. It was shown that the intravenous (i.v.) administration of a recombinant rhesus mAb against α_4_β_7_ (α_4_β_7_ mAb, clone Act-1) to rhesus macaques (RM) just before and during acute i.v. or intrarectal SIV infection leads to lower gut VLs and protects CD4 in the periphery and in the gut in the absence of disease progression [18, 19]. Importantly, administration of the same anti-α_4_β_7_ mAb (herein called, RM-Act-1 to distinguish it from fluorescently labeled mouse Act-1 used for α_4_β_7_ detection by flow cytometry) was recently shown to prevent SIV infection via the vaginal route [19]. Specifically, i.v. administration of 50mg/Kg of RM-Act-1 just before and during an intravaginal repeated low-dose challenge (RLDC) study prevented SIVmac251 infection in 6/12 RMs and, when infection did occur, RM-Act-1 delayed plasma viremia and protected the GALT. A number of mechanisms for the prevention of transmission have been proposed and include the ability of the mAb to inhibit viral spread by preventing the homing of infected α_4_β_7_ CD4^+^ T cells to the GALT and the ability of RM-Act-1 to interfere with the gp120-α_4_β_7_ interaction at the primary site of infection [19]. Of note, a humanized form of the RM-Act-1 mAb is an FDA approved therapy for ulcerative colitis and Crohn’s Disease (Vedolizumab) [20, 21]. Vedolizumab (as well as RM-Act-1 and the original mouse version [22]) binds to a conformational epitope that is unique to the heterodimerized form of the α_4_ and β_7_ chains [23]. Together with humanized mAbs, several anti-integrins small molecules are under various stage of development [24–26]. Among them there is a family of α_4_β_1_/α_4_β_7_ dual inhibitors, which were synthetized and characterized by scientists at Élan Pharmaceutical [24]. These molecules have good oral bioavailability and have shown efficacy in rat models of rheumatoid arthritis (RA) and Crohn’s disease (CD). Unlike mAbs, small-molecule agents can more easily be formulated for topical and oral administration and they can be more easily modified to obtain the desired pharmacokinetics (PK). Since it was shown that the anti-α_4_β_7_ mAb RM-Act-1 could reduce susceptibility to infection when given intravenously, we selected one of Élan’s small molecule anti-α_4_β_7_ inhibitors (compound 12d in [24], here called ELN) to test its effect via the oral and the vaginal routes of administration using a repeated low-dose challenges model of SHIV-SF162P3 infection.

We found that neither the oral alone nor the oral combined with intravaginal administration of ELN, were able to reduce susceptibility to infection, although it decreased the SHIV-associated inflammation in the animals that became infected. Moreover, in order to understand why ELN did not protect, while RM-Act-1 did, we compared the effect of ELN and RM-Act-1 side-by-side in vitro on α_4_β_7_ activation. Notably, integrins can exist in different conformational states, or activation states, that affect ligand recognition and signal transduction [27, 28]. Integrin activation leads to bidirectional signaling crucial in a variety of cellular events such as adhesion, migration, polarity and a series of other physiological changes [29]. We found that, while ELN induces the high-affinity/active conformation of α_4_β_7_, Act-1 inhibits its activation not simply by steric hindrance as previously hypothesized [23], but by stabilizing an inactive or semi-active conformation, thus reducing the ability of the receptor to be activated in the presence of ligands and/or cations. Our results, while shedding light on the role of α_4_β_7_ in HIV and SIV infection, are highly relevant also to the design of anti-integrins therapeutic strategies in autoimmune diseases and particularly in IBD.

## Results

### ELN inhibits MAdCAM-1 and gp120 binding to α_4_β_7_ in vitro

**Fig 1:**
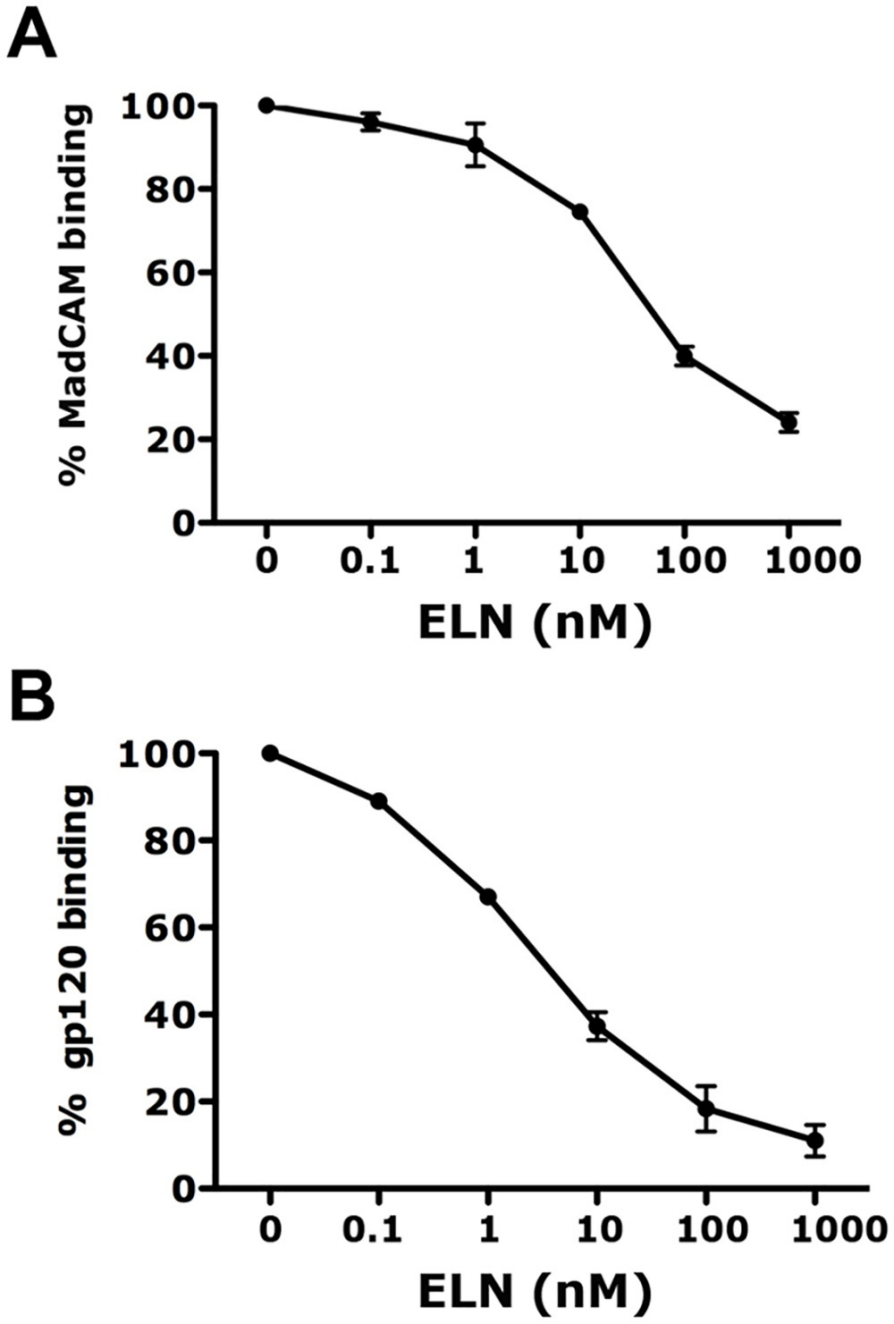
ELN blocks MAdCAM-1 and gp120 binding to α_4_β_7_. Isolated human CD4^+^ T cells were activated with okt3/IL-2 and treated in RA/IL-2 for 5–7 days. A) LIVE/DEAD Aqua stained cells were incubated with indicated concentrations of ELN or mock treated with DMSO, before Neutravidin-PE/Biotin-MadCam-Fc addition. The % of MadCam-PE binding was calculated dividing the frequency of MadCAM-PE positive cells for each condition by the frequency of MadCAM-PE positive cells for the mock condition x100. Mean ± SEMs of 2 experiments are shown. B) LIVE/DEAD stained cells were incubated with anti-CD4 (Leu3A) mAb and indicated concentrations of ELN or mock solution, before Neutravidin-PE/Biotin-gp120 addition. The % of gp120 binding was calculated by dividing the MFI of each condition by the MFI of the mock condition x100%. One of 3 representative experiments is shown. Mean ± SEMs of 3 experiments are shown.

Compound 12d (here referred to as ELN) is a small molecule belonging to a class of compounds that bind to integrin α_4_, developed by Élan Pharmaceuticals and provided to us free of charge for these studies [23][30]. ELN is a α_4_β_1_/α_4_β_7_ dual inhibitor with respectable potency in blocking α_4_β_7_ binding in MadCAM-1 adhesion assays and high oral availability (by cassette AUC in rats). It showed efficacy in the HLA-B27 rat model of IBD and no toxic effects [24]. ELN was selected also because of the relatively large quantities available for in vivo studies and because it was shown to be safe in a Phase I clinical trial. In order to confirm ELN’s ability to interfere with MadCAM-1 binding to α_4_β_7_, we used human CD4^+^ T cells cultured in 100nM retinoic acid (RA) for 5–7 days to increase α_4_β_7_ expression. Indeed, we found a dose-dependent inhibition of the binding of biotinylated soluble MAdCAM-1 to RA-treated CD4^+^ T cells in 1mM MnCl_2_/100μM CaCl_2_ buffer (Mn^++^/Ca^++^ buffer; Fig 1A). Since it has been proposed that direct interaction of HIV-gp120 with α_4_β_7_ may constitute an advantage during transmission [10, 19, 31], although understanding the role of this interaction in HIV transmission was not a primary aim of this study, we tested if ELN could interfere with gp120 binding to α_4_β_7_. We performed an experiment similar to that used to demonstrate inhibition of MAdCAM-1 binding using RA-treated primary CD4^+^ T cells and soluble biotinylated SF162gp120. Indeed, we found that the IC_50_ of ELN for gp120- α_4_β_7_ binding in presence of the αCD4 mAb Leu3A is lower than 10nM (Fig 1B). Anti-α_4_ antibodies have only a modest effect on HIV infection in vitro ([32–34] and our own experience), thus, not surprisingly ELN did not consistently inhibit HIV_SF162_ and SHIV-SF162P3 infection in vitro (S1 Fig).

### Effects of oral and intravaginal ELN administration

**Fig 2:**
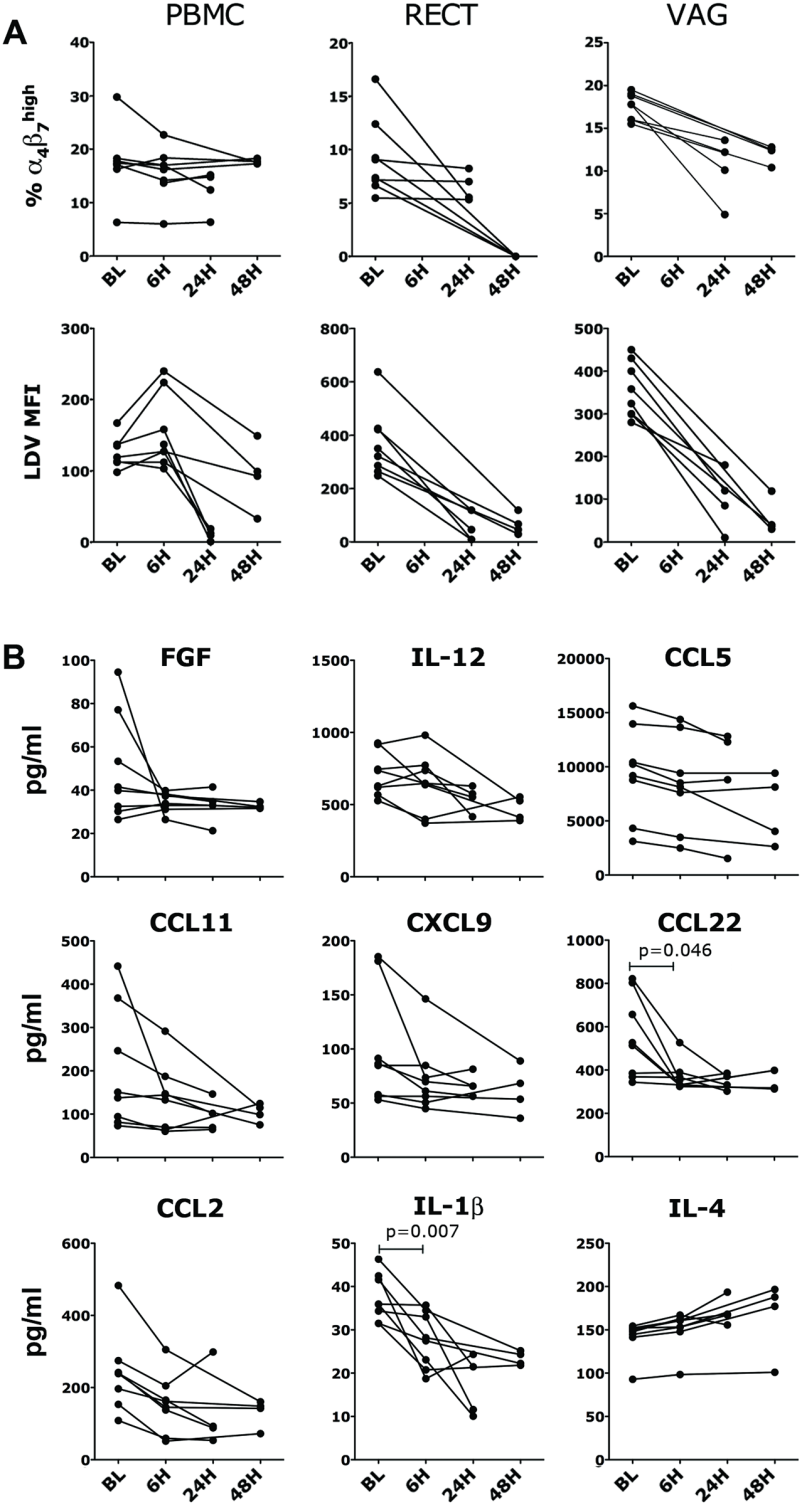
Orally delivered ELN is absorbed and reduces systemic levels of inflammatory factors. 20mg/Kg of ELN was given by oral gavage to 8 animals. All animals were sampled at baseline (BL) and 6h and 4 were sampled at 24h and 4 at 48h. A) Cells from indicated tissues were isolated and stained for flow cytometry. The frequency of α_4_β_7_^high^ cells within live, singlets, CD3^+^ CD4^+^ T cells before and after ELN treatments are shown in the top panel for each compartment. The MFI of the FITC-LDV peptide on α_4_β_7_^+^ CD4^+^ T cells in the same compartment is plotted in the bottom panel. B) The concentrations of soluble factors in blood significantly modulated by ELN treatment are shown. The pre-post treatment differences in each group (24h and 48h) are not significant (Wilcoxon t-test two-tails α<0.05) unless the groups are pooled together (except PBMC; p<0.01), LDV-MFI (p<0.01) and all the CC/CK shown, but for IL-4 (p<0.05).

We hypothesized that interfering with trafficking of α_4_β_7_^high^ CD4^+^ T cells to and from the mucosal site of SIV exposure, could reduce susceptibility to SIV acquisition. In order to test the ability of ELN to inhibit trafficking of α_4_β_7_^+^ cells in vivo, we administered ELN orally to macaques and collected samples at different time points to determine the kinetics of ELN binding to CD4^+^ T cells in different compartments. Eight animals were given 20mg/Kg of ELN by oral gavage. This amount was chosen based on Élan’s PK studies in rodents. However, due to ELN’s poor water solubility only about 70–80% of the drug reached the stomach with this system (we estimated around 100mg/macaque). Blood and tissue samples (vaginal and rectal) were collected at baseline from all animals and at 24h and 48h in four macaques per time point. Blood was collected from all animals also 6h post-treatment. The frequencies of α_4_β_7_^+^ CD4^+^ T cells in blood and tissues were measured by staining with a fluorescently labeled anti-α_4_β_7_ mouse mAb (clone Act-1). As expected, oral ELN had no impact on the frequency of total α_4_β_7_^+^ and α_4_β_7_^high^ CD4^+^ T cells in blood (Fig 2A and gating strategy in S2A Fig). However, ELN substantially reduced the frequency of α_4_β_7_^high^ CD4^+^ T cells in the rectal tissues 2 days after administration and partially reduced their frequency in the vaginal tissue starting 1 day after administration (Fig 2A upper row and S2C Fig). To measure ELN receptor occupancy on CD4^+^ T cells, we used a FITC conjugated LDV (LDV-FITC) peptide that recognizes the main binding site on integrin α_4_ and competes with the binding of ELN (also an LDV-mimetic) on both α_4_β_7_ and α_4_β_1_. The binding of a standardized quantity of FITC-LDV (100nM) decreases proportionally with increased presence of ELN on the surface of the cells (S2B Fig). We verified that, after the oral administration of ELN, the binding of LDV-FITC to CD4^+^ T cells decreased in all tested compartments (Fig 2A, lower row). In blood, ELN occupied all the α_4_β_7_ receptors on CD4^+^ T cells after 24h, but some of the receptors were already free of drug after 48h, while the drug was more stably bound to the α_4_β_7_^+^ cells still present in the tissues up to 48h post-administration. We also determined the effect of ELN on immune factors released systemically and we found evidence of anti-inflammatory properties of the drug. In fact we found a tendency toward decreased concentration of FGF, IL-12, CCL5, CCL11, CXCL9, CCL22, CCL2 and IL-1β after the treatment, with a small increase in IL-4 in the samples from the animals that we tested (Fig 2B). Differences in these factors were significant (p<0.05) when the samples at 24h and 48h post-treatment were grouped in a pre vs post-treatment analysis. A decrease in the levels of IL-17, IL-12, MIF and CXCL11 was present also in the vaginal and rectal fluids of the treated macaques (S3A Fig Also IL-1β and IL-8 were reduced in the vaginal fluids).

**Fig 3:**
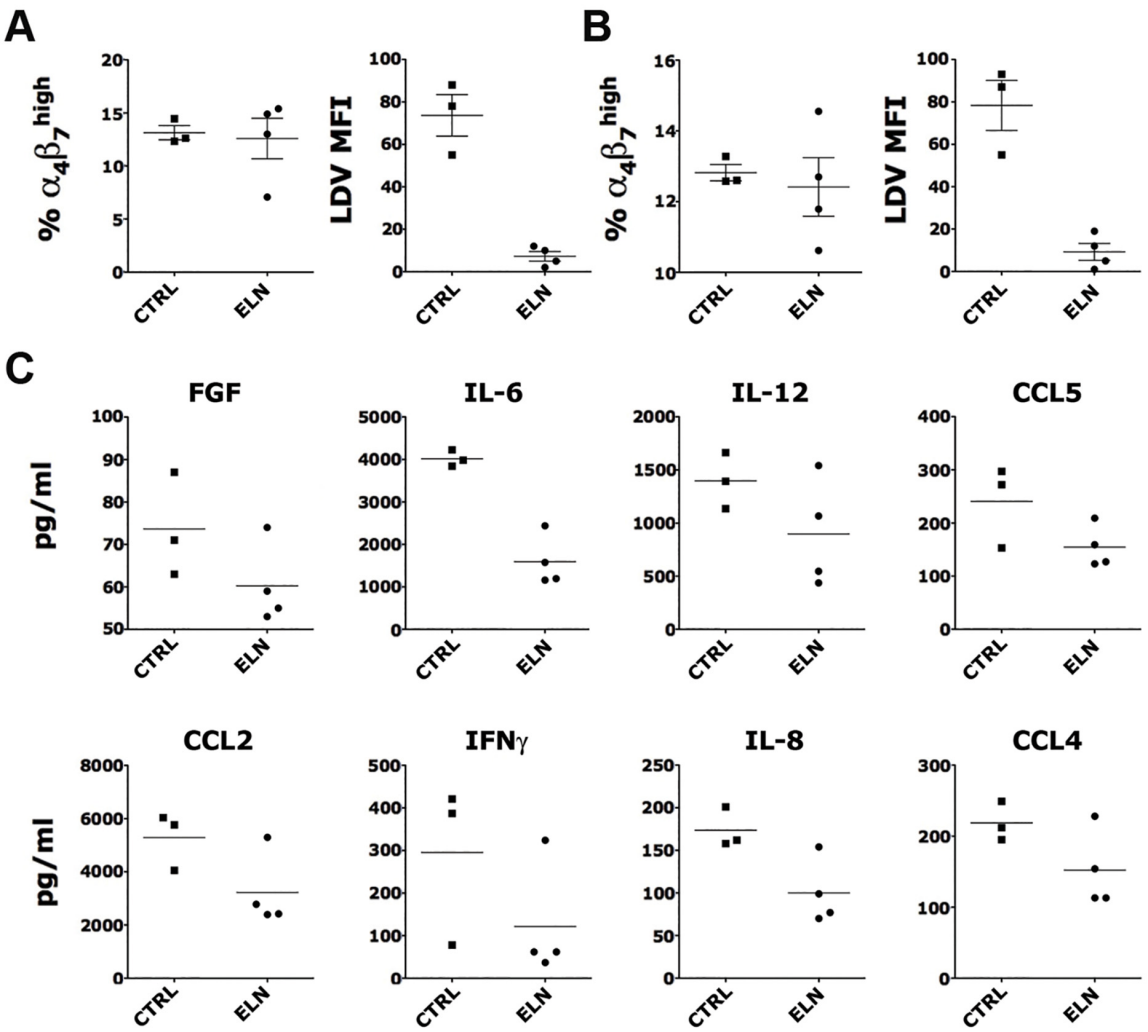
ELN delivered vaginally binds α_4_β_7_ on tissue CD4^+^ T cells and inhibits vaginal inflammatory factors. Two ml of a 0.35% ELN gel were applied within the vaginal cavity of 4 animals and 2ml of placebo gel to 3 animals. A-B) The frequency of α_4_β_7_^high^ cells within live, singlets, CD3^+^ CD4^+^ T cells and MFI of LDV fluorescence of α_4_β_7_^+^ CD4^+^ T cells are shown for vaginal (A) and rectal (B) tissues 4h (vaginal) and 24h (rectal) after gel application. C) The concentrations of soluble factors modulated by ELN treatment in vaginal swabs 4h after gel application are shown. Bars represent mean ± SEM

Since oral administration did not result in a complete masking of the α_4_β_7_ receptors on CD4^+^ T cells in the vaginal tissue, we tested the effect of ELN delivered vaginally. We tested a 0.35 wt. % gel, which was the highest stable concentration of ELN that was possible to formulate in a HEC gel. 2 mL of ELN-gel were applied within the vaginal cavity of 4 animals and 2ml of placebo gel were applied to 3 animals. Vaginal samples were taken 4h post-gel application; and rectal samples 24h post-gel application. As expected from this topical application, we found no differences between the ELN and placebo group in the frequency of α_4_β_7_^+^ CD4^+^ T cells in the vaginal and rectal tissues (Fig 3A and 3B left). However a decrease in the binding of the LDV-FITC indicated that ELN was capable to be absorbed through the vaginal mucosa and mask completely the relevant epitope at the surface of α_4_β_7_^+^ CD4^+^ T cells in both vaginal and rectal compartments (Fig 3A and 3B right). Evaluation of the soluble factors released in the vaginal vault 4h after the gel application suggested that vaginal delivery of ELN also results in an anti-inflammatory effect with a tendency toward decreased levels of several major inflammatory factors in the vaginal fluids (Fig 3C).

### ELN does not inhibit susceptibility to SHIV-SF162P3 vaginal infection

**Fig 4:**
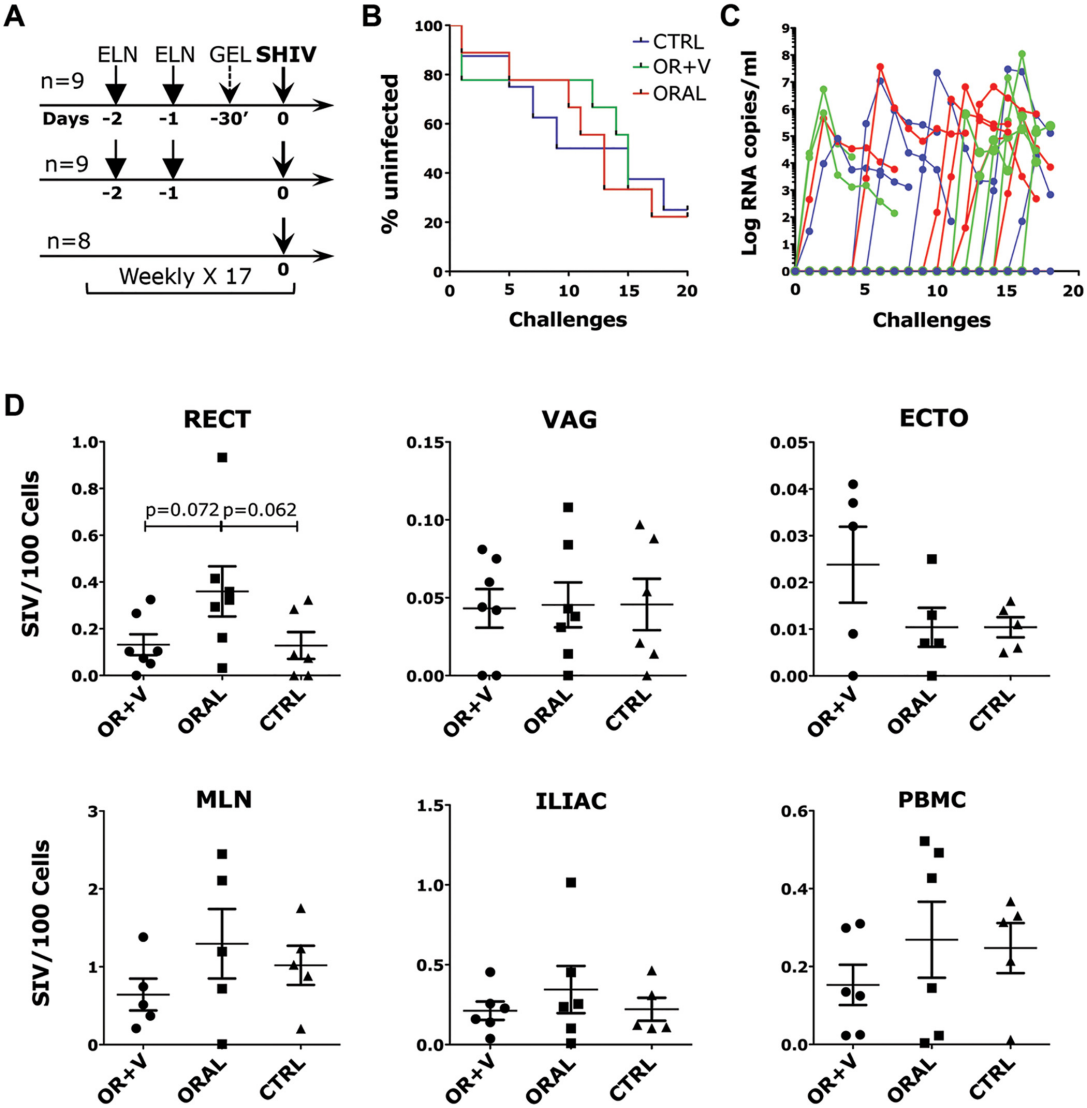
ELN does not reduce susceptibility to SHIV-SF162P3. A) Schematic representation of the study design. Twenty six macaques were divided in 3 groups. Group 1 monkeys received 1 capsule of 100mg of ELN orally for 2 consecutive days and 2ml of a 0.35% ELN gel 30 mins before challenge (n = 9 OR+V group); Group 2 monkeys received 1 capsule of 100mg of ELN orally for 2 consecutive days and 2ml of a placebo gel (n = 9 ORAL group). The final Group received an empty capsule and placebo gel (n = 8 CTRL). This procedure was repeated weekly over 17 weeks or until SHIV infection was acquired. B) Kaplan-Meier curve of the number of challenges required to infect the animals in each group. The challenge performed 1 week before SHIV was detected for the first time in plasma was considered as the infectious challenge. C) SHIV plasma viral loads (VLs) for the first 8 weeks p.i. (until necropsy) D) Cell associated SHIV loads in tissues (RECT, rectal; VAG, vaginal; ECTO, ectocervix; MLN, mesenteric lymph node; ILIAC, iliac lymph node and PBMC) at necropsy. Bars represent mean ± SEM. p<0.05 is considered significant. p<0.125 are shown to indicate tendency toward significance (Mann-Whitney).

**Fig 5:**
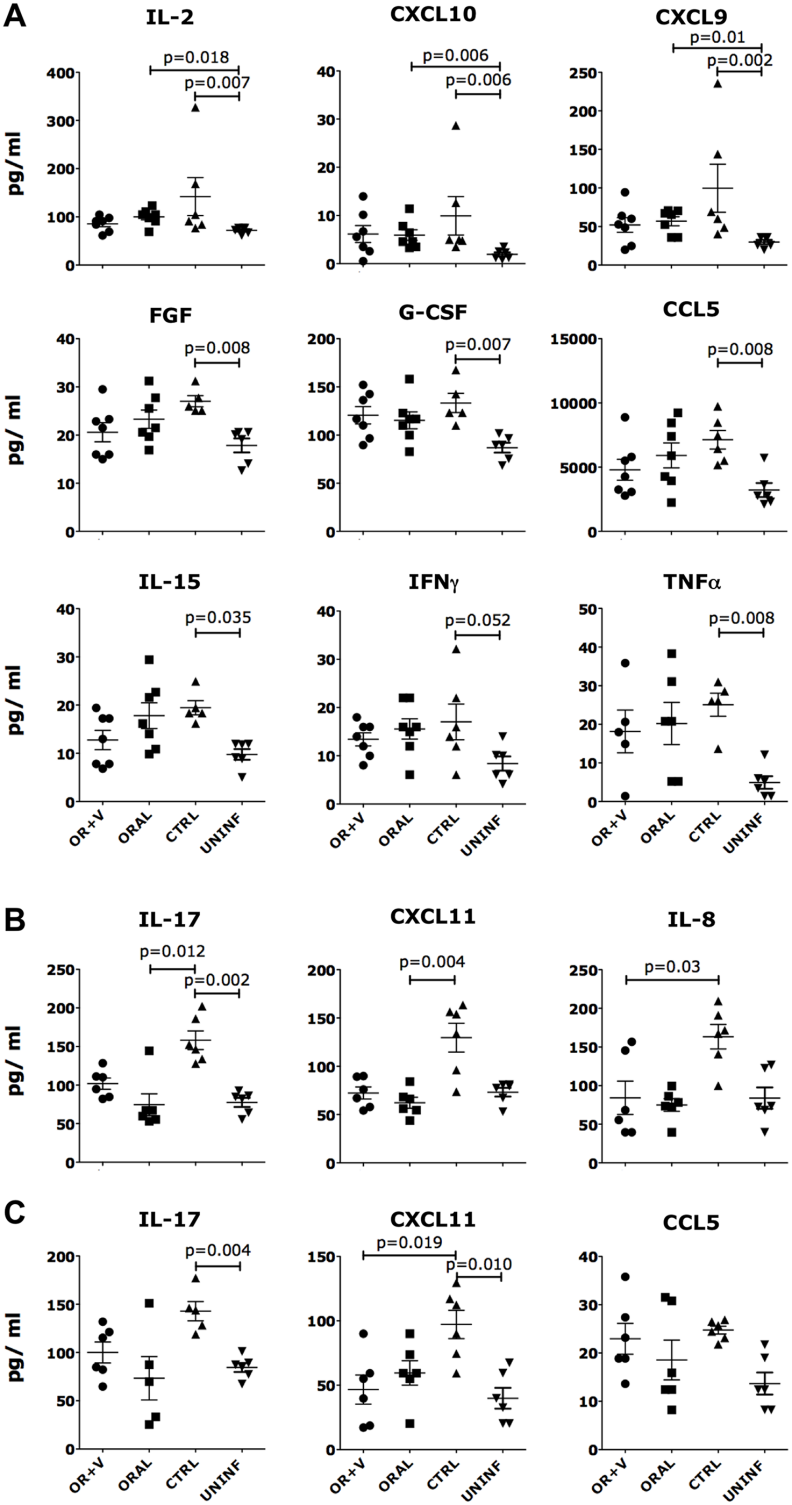
ELN reduces SHIV-induced inflammatory status. A) Concentration of soluble factors significantly (Kruskal-Wallis H test) modulated by SHIV infection in control animals (vs uninfected/untreated, n = 6) in the plasma of SHIV infected macaques ~3 weeks post-infection. (B-C) Concentration of soluble factors significantly (Kruskal-Wallis H test) modulated by ELN in vaginal (B) and rectal (C) fluids collected at necropsy. The factors modulated in blood at necropsy are shown in S4 Fig. Bars represent mean ± SEM. Mann-Whitney p values are shown as post-hoc pairwise analysis for all the factors significantly different by Kruskal-Wallis (p<0.05 is considered significant).

Since our PK studies indicated that orally administered ELN would bind all α_4_β_7_ receptors on blood CD4^+^ T cells at 24h, but that the biggest impact in tissues would be 48h after administration, for the anti-SHIV in vivo study we decided to administer the drug orally for 2 consecutive days and then challenge 24h after the second dose. Moreover, since using oral gavage it was impossible to control for the amount of ELN lost due to its insolubility in water and adhesiveness to the tube, we used gel capsules filled with 100mg (estimated amount delivered by oral gavage in the PK study) of ELN. The capsules were placed directly in the stomach of the macaques with an oral cannula. Since we had demonstrated the ability of ELN to block trafficking of α_4_β_7_ CD4 T cells to the rectal and vaginal compartments in vivo, we decided to test the effect of ELN on susceptibility to a repeated regimen of vaginal SHIV-SF162P3 challenges. To this end, we devised a three-arm study including a group with ELN delivered only orally (n = 9), a group in which vaginal delivery (2ml of the 0.35% gel) was added to the oral delivery 30’ before each challenge to increase coverage of the α_4_β_7_ receptors within the vaginal tissue at the time of exposure (n = 9) and a control group with only placebo treatment (n = 8; Fig 4A). The first 5 weekly challenges were performed with 100 TCID_50_ of SHIV-SF162P3 and the remaining 12 challenges with 200 TCID_50_. Challenges and treatments were stopped after infection was confirmed by detection of virus in blood for 2 consecutive times, each one week apart. There was no significant difference in the acquisition of infection among the groups (Fig 4B), neither was there any difference in the acute plasma VL (Fig 4C). 7/9 animals became infected in the oral+ vaginal treatment group, 7/9 in the oral treatment group and 6/8 animals became infected in the control group. The infected macaques were euthanized 8 weeks after the first detection of virus in blood and several tissues were collected at necropsy. Tissues were used to determine possible differences in virus distribution as a result of the treatment. We found a tendency toward a higher cell-associated viral load in the rectal tissue and mesenteric LNs (MLNs) of the animals treated with the drug orally, but the difference was not significant (Fig 4D). An analysis of plasma soluble factors during acute infection (time of the second detection of virus in blood, ~3 weeks p.i.) revealed that, while the level of several inflammatory factors was significantly higher in the SHIV-infected animals in the control group compared to uninfected animals, the increase was much lower and non-significant in the SHIV-infected animals from both treated groups (Fig 5A; in all CC/CK shown but for IL2, CXCL10 and CXCL9, where the difference between the oral and group and uninfected was also significant). Instead, when the levels of CC/CK in blood were measured at the time of necropsy (when we could also measure CC/CK in vaginal and rectal fluids), there was no difference in most CC/CK that were different in the acute phase of infection between the infected macaques in the control group and the uninfected macaques (S4 Fig). The difference was still significant only in IL-15 and CCL5 (although significant differences were now found in IL-1RA and CCL11). No significant differences were present between the infected macaques in the treatment groups and the uninfected macaques also at necropsy (S4 Fig). Moreover, in the vaginal swabs at necropsy we found that the levels of IL-17 and CXCL11 were significantly lower in the orally treated animals compared to the control group, while IL-17 was significantly higher in the control group compared with uninfected animals in both vaginal and rectal fluids (Fig 5B and 5C). Finally, the levels of CXCL-11 in the rectal fluids and IL-8 in the vaginal fluids were significantly lower in the orally and vaginally treated group than in the controls. Although the anti-inflammatory effect of ELN was not sufficient to prevent SHIV acquisition, overall these results indicate that ELN tends to dampen the SHIV-induced inflammatory status.

**Fig 6:**
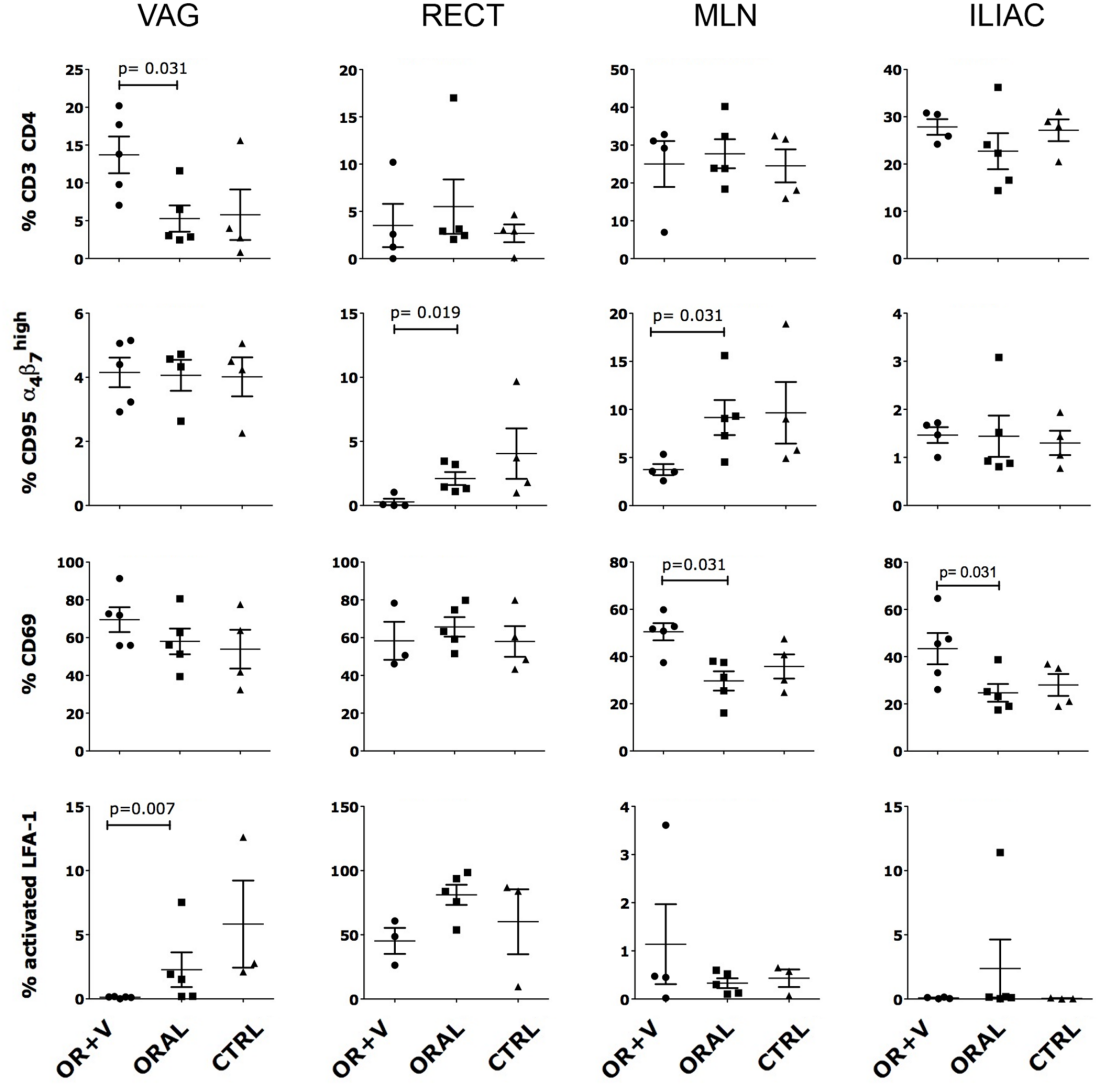
ELN modulates CD4^+^ T cells phenotype in SHIV infected macaques. Isolated cells from indicated tissues at necropsy were stained with LIVE/DEAD Aqua and an antibody combination of anti-: CD4, CD3, α_4_β_7_, CD69, CD95, CCR5 and LFA-1 (clone MEM148, which recognizes only the activated form). The frequency of cells expressing the indicated marker within live, singlets, CD3^+^ CD4^+^ T cells (within CD95^+^ for the α_4_β_7_^high^) are shown. Bars represent mean ± SEM. p<0.05 is considered significant (Mann-Whitney).

Although ELN treatment during viral exposure did not protect from SHIV acquisition, we hypothesize that it may have changed cell distribution and trafficking so to impact the differential depletion of activated CD4^+^ T cell subsets. Interestingly, an analysis of the phenotype of CD4^+^ T cells in different compartment at the time of necropsy revealed that the animals treated both orally and vaginally had significantly lower depletion of CD4^+^ T cells in the vaginal tissue than the orally treated group and a similar tendency was present when compared with the control group (Fig 6). This is despite similar levels of SIV tissue viral loads in the vaginal compartment among the treatment groups (Fig 4D). Differences in trafficking of infected cells due to the ELN treatment and reduced apoptosis due to ELN anti-inflammatory effect may contribute to explain these seemly contradictory results. The oral + vaginal treatment had also a significant impact on the frequency of α_4_β_7_^high^ and CD69^+^ CD4^+^ T cells in the mesenteric lymph nodes and it significantly reduced the frequency of CD4^+^ T cells expressing the activated form of LFA-1 in the vaginal tissue compared to the group treated only orally (Fig 6). A non-significant higher depletion in the frequency of CCR5^+^ CD4^+^ T cells was also seen in both treated groups in the vaginal tissue and iliac LNs (S5 Fig).

### ELN activates α_4_β_7_, while Act-1 inhibits its activation

**Fig 7:**
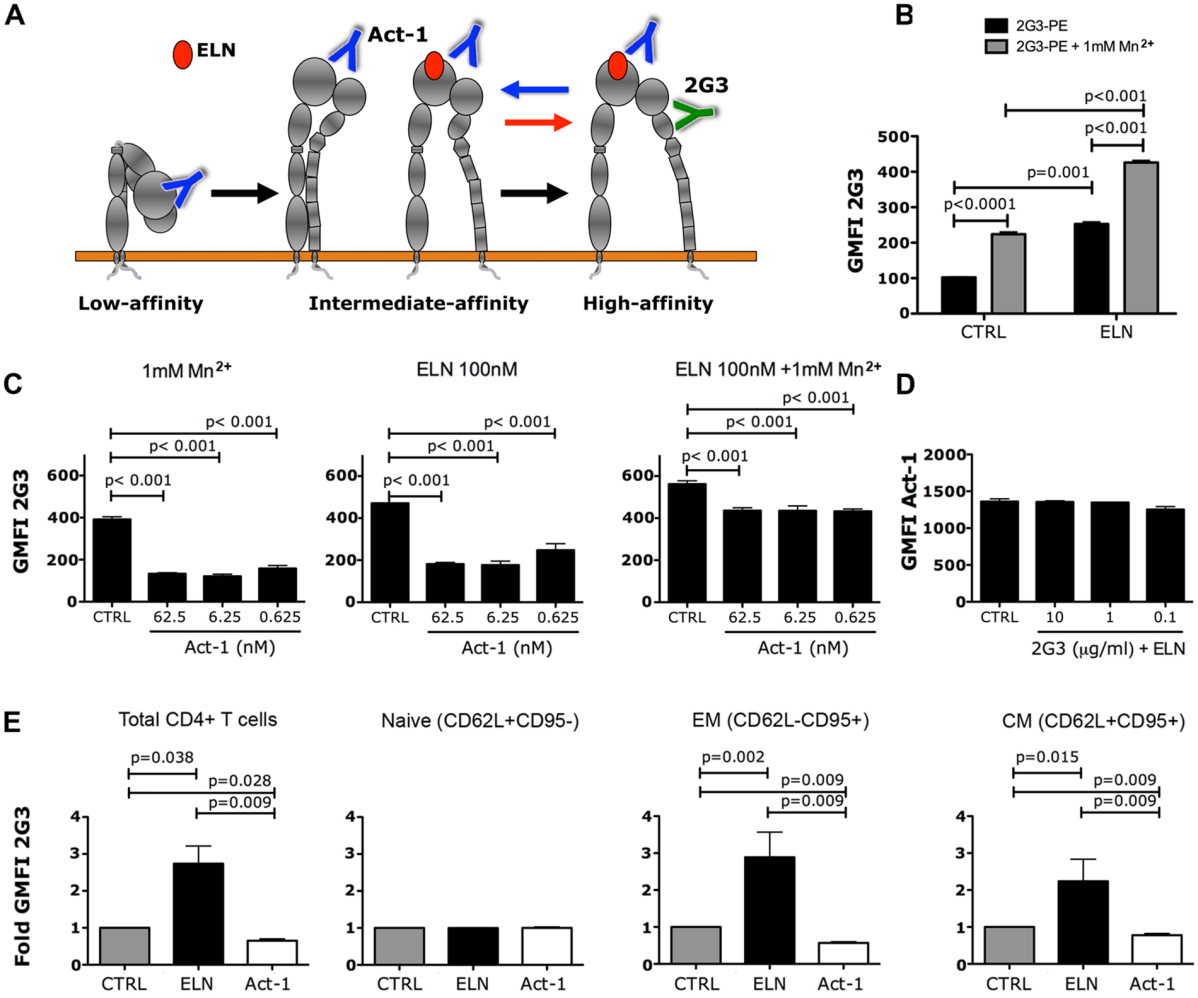
ELN induces, while Act-1 inhibits, α_4_β_7_ active conformation. (A) Binding of ELN, Act-1 and 2G3 to α_4_β_7_ and proposed effect on its conformation. (B-C) RPMI8866 cells plated in Ca^2+^/Mg^2+^ assay buffer were incubated with ELN (B) or Act-1 (C) at indicated concentrations. Cells were then stained with PE-conjugated 2G3 Ab +/- Mn^2+^ (1mM) and ± ELN in (C). Geometric MFI (GMFI) of 2G3-PE was plotted. (D) RPMI8866 cells in Ca^2+^/Mg^2+^ were incubated with increasing concentrations of unlabeled 2G3 + ELN at 100nM. Cells were then stained with PE-conjugated Act-1 mAb and MFI of Act-1-PE is shown. (E) Similar experiment as described in (B) but using RA-cultured CD4^+^ T cells and adding CD95 and CD62L mAbs for memory subsets phenotyping (EM, effector memory; CM, central memory). Results were expressed as fold change GMFI after normalization to their respective control (set as 1). Each bar represent mean of at least 5 independent experiments/donors ± SEM. P< 0.05 is considered significant.

The intravenous injection of Act-1, an anti-α_4_β_7_ antibody, successfully reduced susceptibility to vaginal SIVmac251 infection in repeated low-dose challenges studies [19]. This antibody mainly masks the β_7_ subunit on its β-propeller domain [23] and competes efficiently with MadCAM-1 and gp120 binding [19] as ELN does (Fig 1). In order to understand the differential effect of Act-1 and ELN on SIV/SHIV acquisition in vivo, we dissected more in depth their effect on CD4^+^ T cells and on the α_4_β_7_ receptors in vitro, comparing them side-by-side. Using an antibody (clone 2G3), which is able to recognize only the activated form of integrin α_4_β_7_, we were able to discriminate the effect of the 2 compounds on the receptor (Fig 7A). The 2G3 mAb specifically recognizes a Mn^2+^- and ligand-induced epitope on β_7_. Its binding is increased by MAdCAM-1 and reduced by Ca^2+^ and it is considered an integrin-activation reporter [35, 36]. We first evaluated the effect of ELN and Act-1 on 2G3 binding to RPMI 8866 cells. This B lymphoma cell line expresses high levels of α_4_β_7_, through which it can bind to MAdCAM-1 even in the absence of Mn^2+^ [37]. However, binding of 2G3 to RPMI 8866 was very poor in the absence of Mn^2+^ and it was substantially increased in the presence of 1mM Mn^2+^ or 100nM ELN alone or further increased in the presence of both ELN and Mn^2+^ (Fig 7B and dose-response curve in S6A Fig).

**Fig 8:**
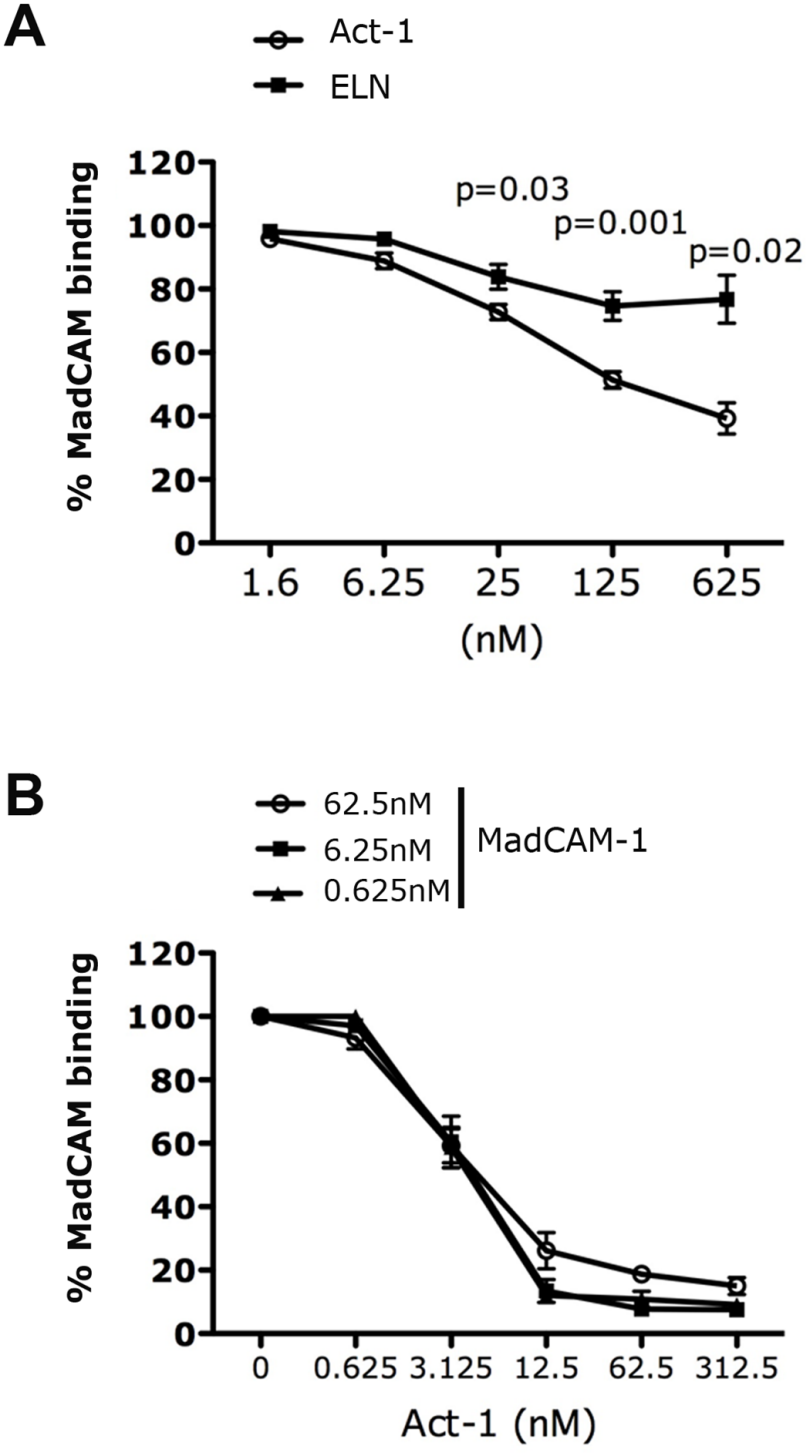
Act-1 acts as an allosteric inhibitor. (A-B) LIVE/DEAD stained RA-cultured CD4^+^ T cells in Mn^2+/^Ca^2+^ were incubated with recombinant MadCAM-Fc-biotin (0.1μg in (A) and 10 fold increasing concentrations in (B). In (A) MadCAM-Fc was first added and wash. Then indicated concentrations of unlabeled Act-1 or IgG1 or ELN were added. In (B) MadCAM-1 was added at the same time with various concentrations of unlabeled Act-1. Results were expressed as % of MadCAM-1 binding after normalization to their respective control (set as 100). Each bar or symbol represent mean ± SEM of n = 8 (A) and n = 4 (B) independent experiments.

In contrast, when similar experiments were performed using Act-1, 2G3 binding in the presence of either Mn^2+^ or ELN was significantly reduced by Act-1 (Fig 7C left and center and dose-response curve in S6B Fig). This reduction was less pronounced when integrin activation was forced with the presence of both Mn^2+^ and ELN (Fig 7C, right). The 2G3 binding reduction suggests that Act-1 may inhibit α_4_β_7_ activation or stabilize the integrin in its semi-active, intermediate conformation, partially masking the 2G3 epitope. In order to ensure that these effects of ELN and Act-1 were relevant in primary cells, these experiments were repeated on isolated human CD4^+^ T cells cultured in RA. Indeed, we confirmed that ELN is able to increase 2G3 binding to CD4^+^ T cells, while Act-1 decreases its binding. Of note, the effect is more pronounced in cells with an activated memory-like phenotype, but not in naïve cells (Fig 7E). To exclude the possibility that the reduced binding of 2G3 in the presence of Act-1 is due to steric inhibition, we studied the ability of the 2 mAbs to compete for the same epitope, measuring the binding of Act-1-PE to RPMI8866 cells in presence of increasing concentration of unlabeled 2G3 +/- ELN. We found that Act-1 binding is not affected by 2G3 binding in the presence Mn^2+^ or in the presence of both Mn^2+^ and ELN (Fig 7D and in the absence of ELN, S7 Fig). This indicates that Act-1 and 2G3 do not compete for the same epitope and suggests that Act-1 may act as allosteric inhibitor. In order to investigate this possibility, we first tested the ability of Act-1 to displace pre-bound MAdCAM-1, comparing it with ELN. We found that Act-1 displaces MAdCAM-1 with an IC_50_ of ~62 nM, while ELN is rather inefficient at dissociating MadCAM-1 with less than 15% dissociation at 625nM (Fig 8A). Finally, we demonstrated allosteric inhibition in co-addition competition studies, where different concentrations of MAdCAM-1 and Act-1 where added at the same time in the presence of 1mM Mn^2+^. We found that the amount of Act-1 required to inhibit MAdCAM-1 binding is the same over a 10-fold range of MadCAM-1 concentrations (IC_50_ = 1.2μg/ml for 10μg, 1.8μg/ml for 1μg and 1.6μg/ml for 100ng; Fig 8B). In addition, the maximal extent of inhibition decreased with increasing MadCAM-1 concentration. If Act-1 behaved as a direct competitive inhibitor of MadCAM-1 binding, the concentration of Act-1 for half maximal inhibition of binding should increase in parallel with the MAdCAM-1 concentration, and the maximal extent of inhibition should be unchanged. Instead, these results are consistent with an allosteric inhibition, in which Act-1 does not directly compete with MadCAM-1 for binding to the β7 subunit, but binds to a separate site and decreases the affinity of MadCAM-1 binding by an allosteric effect on the conformation of the integrin. Moreover, we found that even in the presence of saturating concentration of Act-1-APC bound to RA-treated CD4^+^ T cells, a substantial amount of MAdCAM-1 binding is still detected in the absence of Act-1 displacement (S8 Fig). This further supports the notion that Act-1 is not sterically masking epitopes important for MAdCAM-1 binding, although it may still be masking epitopes important for firm adhesion.

**Fig 9:**
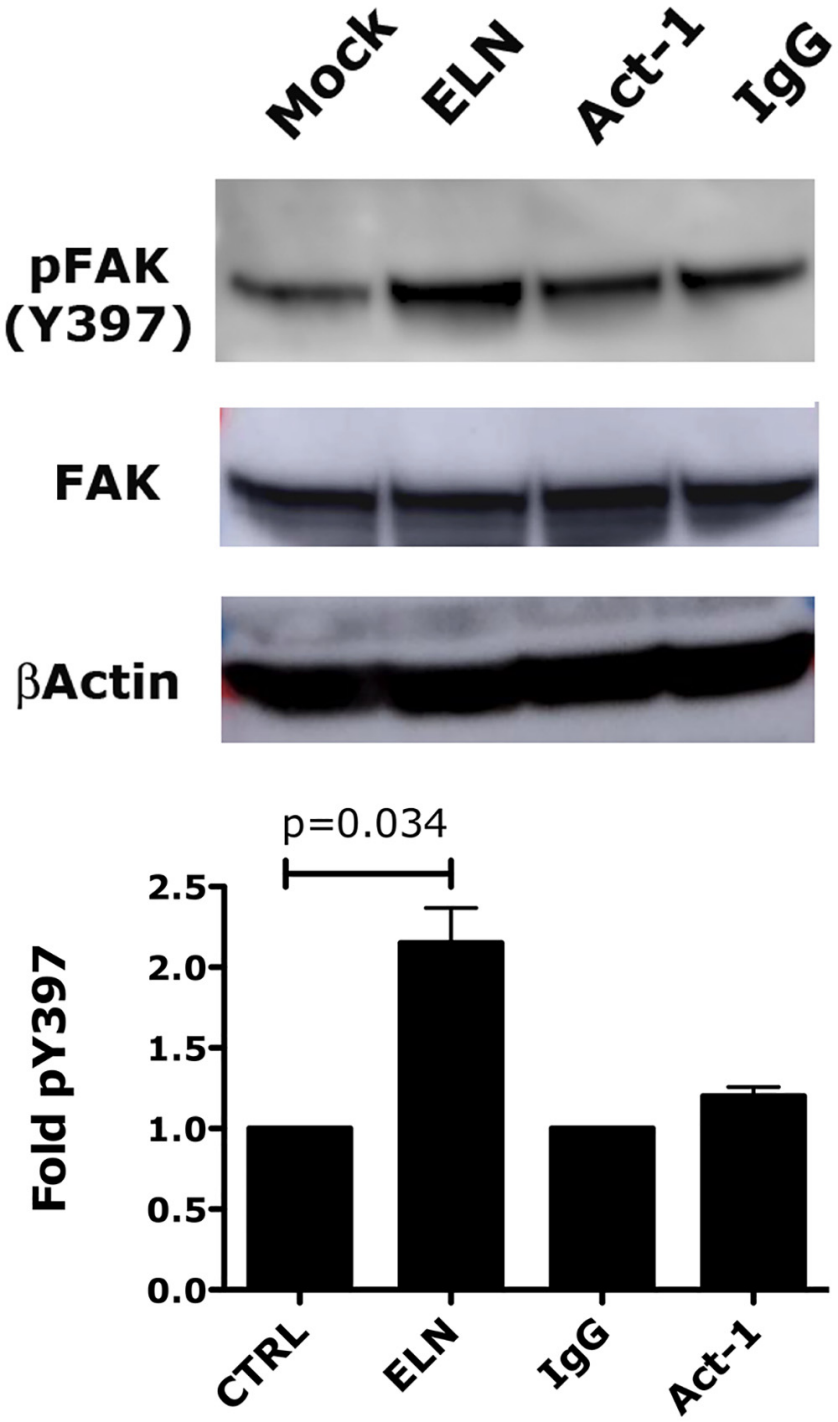
ELN increases FAK phosphorylation. RA-treated CD4+ T cells or Hut78 cells (S9 Fig) were incubated with ELN (1μM), Act-1 (500nM), IgG1 (500nM) or control (mock treated with DMSO) for 15 mins at 37°C on fibronectin coated wells. Cells were lysed and phospho-Y397-FAK, FAK and β actin expression were determined by western blotting. Relative protein expression was determined for pFAK and FAK using ImageJ. The data were normalized against FAK expression. Top panel: one representative blot is shown for CD4+ T cells. Bottom panel: results from 3 independent experiments done with CD4+ were expressed as fold change in pY397 FAK after normalization to their respective control (set as 1). Mean ± SEMs are shown. p< 0.05 is considered significant.

Finally, integrin stimulation by natural ligands initiates an intracellular signal that involves the autophosphorylation of Focal Adhesion Kinase (FAK) Y397 [38, 39]. We found that while ELN increases the amount of phosphorylated Y397 in RA-treated CD4+ T cells and Hut78 treated with fibronectin, Act-1 did not (Fig 9).

### ELN increases LFA-1 activation in vivo

**Fig 10:**
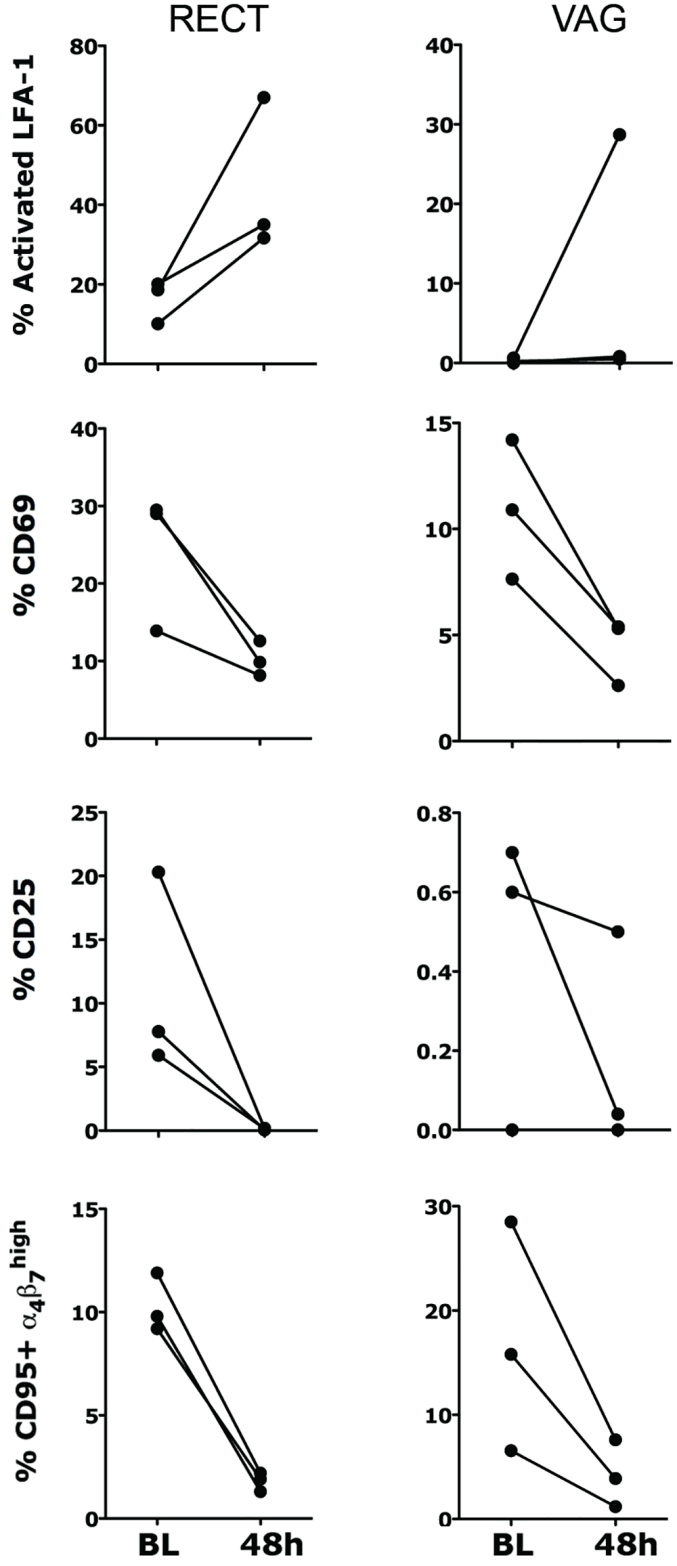
ELN modulates cell phenotype in vivo. 3 uninfected animals were administered a 100mg capsule of ELN for 2 consecutive days. Animals were sampled at BL and 24h after the 2^nd^ administration (48h total). Isolated cells from indicated tissues were stained with a viability dye (DAPI) and an antibody combination of anti-: CD4, CD3, α_4_β_7_, CD69, CD25, CD95, CCR5 and LFA-1 (clone MEM148, which recognizes only the activated form). The frequency of cells expressing the indicated marker within live, singlets, CD3^+^ CD4^+^ T cells are shown.

Since we established that the likely reason for ELN failing to inhibit SHIV infection was its ability to activate the integrin, we sought to determine the potential effect of α_4_β_7_ activation on the phenotype of CD4^+^ T cells in vivo. Although we could not measure any specific effect of ELN (or Act-1) on the expression of CCR5, CD25, CD69, CD95 or CD62L on CD4^+^ T cells (isolated or in mixed PBMC cultures) in vitro (S10 Fig), we hypothesize that the effect could be evident only in tissues after in vivo administration of ELN. In order to prove our hypothesis we administered 100mg capsule of ELN for 2 consecutive days (similar to the protocol that we utilized in the SHIV efficacy study) to 3 animals and we euthanized them 24h after the 2^nd^ administration (time of SHIV-SF162P3 challenge in the efficacy study). Indeed we found that ELN treatment increased the frequency of rectal CD4^+^ T cells expressing the activated form of LFA-1 in these 3 animals. Moreover, a tendency toward a reduced frequency of CD69^+^ and CD25^+^ CD4^+^ T cells in the rectal and vaginal compartments was also detected (Fig 10). The levels of inflammatory CC/CK in blood and vaginal and rectal fluids of these 3 animals also tended to decrease in a way very similar to the decrease found in the initial PD studies, when ELN was given through oral gavage, confirming the similarity of the 2 administrations (S3B and S3C Fig).

## Discussion

α_4_β_7_^high^ cells are a preferential target of HIV and SIV/SHIV infection and they are depleted particularly during the acute stages of the infection [7, 11, 40]. Strategies that target α_4_β_7_ and aim to preserve the CD4^+^ gut-homing T cell populations may be advantageous not only for their potential to reduce the risk of HIV acquisition, but also for their ability to protect the GALT. Indeed the initial depletion of CD4^+^ T cells in the gut may profoundly influence the rate of disease progression [41–43]. However, herein we show that due to the complexity of the interaction between α_4_β_7_ and its ligands, different anti-α_4_β_7_ strategies may lead to very different results. We know that binding of gp120 to α_4_β_7_ is not needed for viral entry or infection and that blocking this interaction by itself has little or no effect on HIV/SIV infection in vitro [11, 32, 33]. Indeed our data are consistent with α_4_β_7_’s role in HIV/SIV transmission as a facilitating factor in virus systemic expansion and not attachment and entry. Our results highlight that whatever effect the interference with the HIV-α_4_β_7_ interaction may have in vitro and in vivo is easily confounded by the cell stimulation driven by the binding of α_4_β_7_ to the agonist/antagonist. We found that even the Act-1 mAb, which protected from SIVmac251 acquisition, may not be simply sterically interfering with the receptor binding to its natural ligand as previously hypothesized by Yu et al. [23], but it appears to impact the activation of the integrin, probably stabilizing a more closed and inactive conformation. Of note, our data on Act-1 do not directly contradict the hypothesis formulated by Yu et al, where superposition of MAdCAM-1 on VCAM-1 docked to the α_4_β_7_-Act-1 EM complex suggested that Act-1 sterically interferes with the binding of MAdCAM-1 charged antenna. In fact, Act-1 might also partially block residues that are important for the firm adhesion of α_4_β_7_ to MAdCAM-1, while allowing and stabilizing a more closed, low-affinity conformation of α_4_β_7_, which can still bind both VCAM and MAdCAM-1 and is perhaps involved in rolling, but not in adhesion. In fact, we have shown that MAdCAM-1 can still bind in solution to α_4_β_7_ bound to saturating concentration of Act-1. On the other hand, ELN induces the active conformation of α_4_β_7_ and transduces a signal in the cells. This cell stimulation may, at the same time, promote HIV infection and explain the anti-inflammatory properties of the drug. The anti-inflammatory activity of ELN should have helped decrease susceptibility to infection, but perhaps, in the context of increased cellular activation/proliferation due to integrin stimulation this effect was not evident. More studies are needed to understand the impact of α_4_β_7_ activation on HIV infection. We did not detect ELN-mediated changes in the expression of CD69, CD25 and LFA-1 in vitro, but we may have missed them or perhaps our assay failed to mimic complex cell-to-cell interactions present in vivo. In fact, we detected changes in the expression of these receptors after oral ELN administration. In particular, there was a tendency for increased LFA-1 activation in the rectum. Stimulation of integrin α_4_ was previously shown to activate LFA-1 in vitro [44] and gp120 interaction with α_4_β_7_ appears also to stimulate LFA-1 activation [33]. Thus, ELN might also have increased viral spread by inducing the formation of virological synapses. However, we cannot exclude that the increase in LFA-1 was the results of systemic effects of changes in immune cell trafficking patterns instead that a direct effect of ELN’s stimulation of α_4_β_7_. We could not detect an effect of ELN alone on cell proliferation, but it is plausible that ELN acts as co-stimuli and exerts an effect on cell proliferation only together with CD3 or CD4 stimulation. Co-stimulation through α_4_β_7_ has indeed been reported [45]. It is possible that the lack of any enhancing or protective effect of ELN on SHIV infection is due to ELN’s opposite effect on factors that can both enhance and decrease susceptibility to SHIV. Indeed, LFA-1 activation may stimulate cell-to-cell contact and viral spread (although this was mostly in the rectal tissue perhaps because of its higher basal expression of LFA-1 and α_4_β_7_), while ELN’s anti-inflammatory activity may inhibit cell activation (decreasing CD69 and CD25), decreasing susceptibility to SHIV and counterbalancing the enhancing effect.

One shortcoming of this work is the lack of data on Act-1’s effect on a RLDC model with SHIV-SF162P3. However, the SHIV-SF162P3 virus was constructed on the SIVmac239 backbone [46] and SIVmac239 and SIVmac251 are closely related cloned and uncloned macaque SIV isolates, with SIVmac251 resulting in a more significant SIV-driven immune activation during the earliest stages after transmission [47]. Since we can exclude that the protective effect of Act-1 is due to its interference with the SIVmac251 gp120-α_4_β_7_ binding (ELN also interferes with SF162 gp120-α_4_β_7_ interaction), it is likely that Act-1’s protection from SIV acquisition in the SIVmac251 model can be translated to the SHIV-SF162P3 model. Finally, we cannot exclude the presence of off-target effects due to ELN ability to bind also α_4_β_1_ (although ELN does not seem to be able to activate α_4_β_1_).

Of note, while anti-α_4_ therapies with humanized mAbs have been relatively successful for the treatment of multiple sclerosis (Tysabry) and IBD (Vedolizumab and AMG181), attempts to develop small-molecules selective α_4_β_1_ or α_4_β_7_ antagonists have failed until now, possibly for the same reasons for which ELN failed to protect against SHIV-SF162P3. Integrins activation and the mechanisms of cell adhesion are extremely complex processes that involve receptors clustering and conformational changes that can be the result of signaling from within the cell and of outside stimuli. Despite the challenges, targeting α_4_β_7_ for HIV prevention remains a promising strategy and more investigations are needed to clarify exactly the mechanisms by which Act-1 protected from SIV acquisition and the role of α_4_β_7_ signaling and interaction with its ligands play in HIV infection.

## Material and Methods

### Ethics statement

A total of 26 female Indian-origin rhesus macaques (Macaca mulatta, RM; mean age: 8.4 years range: 4.9–12 years; mean weight: 8.1kg range: 4.55–11.09 kg) were housed in compliance with the regulations under the Animal Welfare Act, the Guide for the Care and Use of Laboratory Animals, at Tulane National Primate Research Center (TNPRC; Covington, LA). Animals were socially housed, indoors in climate controlled conditions with a 12/12-light/dark cycle. All the animals on this study were monitored twice daily to ensure their welfare. Any abnormalities, including those of appetite, stool, behavior, were recorded and reported to a veterinarian. The animals were fed commercially prepared monkey chow twice daily. Supplemental foods were provided in the form of fruit, vegetables, and foraging treats as part of the TNPRC environmental enrichment program. Water was available at all times through an automatic watering system. The TNPRC environmental enrichment program is reviewed and approved by the IACUC semiannually. Veterinarians at the TNPRC Division of Veterinary Medicine have established procedures to minimize pain and distress through several means. Monkeys were anesthetized with ketamine-HCl (10 mg/kg) or tiletamine/zolazepam (6 mg/kg) prior to all procedures. Preemptive and post procedural analgesia (buprenorphine 0.01 mg/kg) was required for procedures that would likely cause more than momentary pain or distress in humans undergoing the same procedures. The above listed anesthetics and analgesics were used to minimize pain or distress associated with this study in accordance with the recommendations of the Weatherall Report. The animals were euthanized at the end of the study using methods consistent with recommendations of the American Veterinary Medical Association (AVMA) Panel on euthanasia and per the recommendations of the IACUC. Specifically, the animals were anesthetized with tiletamine/zolazepam (8 mg/kg IM) and given buprenorphine (0.01 mg/kg IM) followed by an overdose of pentobarbital sodium. Death was confirmed by auscultation of the heart and pupillary dilation. None of the animals became severely ill or died prior to the experimental endpoint. The TNPRC policy for early euthanasia/humane endpoint was included in the protocol in case those circumstances arose. All studies were approved by the Animal Care and Use Committee of the TNPRC (OLAW assurance #A4499-01) and in compliance with animal care procedures. TNPRC is accredited by the Association for Assessment and Accreditation of Laboratory Animal Care (AAALAC#000594).

### Macaque treatments

8 RMs were used for PK studies to investigate administration of ELN (provided free of charge by Dr. Andrei Konradi and Élan) by oral gavage: ELN (20mg/Kg of monkey weight) was mixed with 10ml of water, although only 70–80% of the drug was fully in suspension and was delivered within the stomach cavity. Blood and tissue samples were collected at 4h, 24h or 48h as indicated in the results section. 7 RMs were used to test intravaginal administration: 4 animals received 2ml of the 0.35% ELN gel and 3 RMs received 2ml of placebo gel applied atraumatically within the vaginal cavity. 0.35% ELN gel was prepared by mixing the required mass of ELN with an established hydroxyethylcellulose (HEC) gel placebo formulation [48]. HEC gel without ELN was used as placebo. Samples were collected at 4h and 24h as indicated in the result section. For the efficacy study, a total of 26 RMs were divided into 3 groups, MHC typed and randomized for the Mamu*A01, B08 and B17 alleles. No depot medroxyprogesterone acetate treatment was performed and the study was performed in the summer months when most of the macaques are not cycling (Data from previous studies. The menstrual cycle of these macaques was not monitored). Each monkey received orally 1 cellulose capsule (NowFoods) manually filled with 100mg of ELN for 2 consecutive days (n = 9 OR+V group, and n = 9 ORAL group) or an empty capsule (n = 8 CTRL) and 2ml of a 0.35% ELN gel (n = 9 OR+V group) or 2ml of placebo gel (n = 9 ORAL and n = 8 CTRL) 30’ before viral challenge (Fig 4A). RMs were then challenged following a RLDC protocol where 100 TCID_50_ of SHIV-SF162P3 in 1 mL of PBS was applied in the vaginal cavity. This procedure was repeated weekly for the first 5 weeks and then the inoculum was increased to 200 TCID_50_ for the remaining 12 weeks or until SHIV infection was confirmed (nested-SIVgag PCR on PBMC were positive for two consecutive weeks). Virus was originally obtained from the NIH AIDS reagent program and propagated and titrated in RMs PBMCs. Infected animals were euthanized 6 weeks after the 2^nd^ detection of virus in PBMC and blood and various tissues, including lymph nodes (mesenteric and iliac) and mucosal tissues (jejunum, rectal, vaginal and endocervix) were collected.

### Cell isolation and flow cytometry

PBMCs were isolated using Ficoll-Hypaque density gradient centrifugation. Mucosal tissues at necropsy were cut in 0.5cm^2^ pieces and incubated 40 mins at RT in HBSS without Ca^2+/^Mg^2+^ with DTT, 1.7mM EDTA and 100μg/ml of Gentamicine on a shaking platform. Pieces were washed in HBSS with Ca^2+/^Mg^2+^ and digested 45 mins in HBSS with 1 mg/mL Collagenase IV (Worthington Biochemical) and 1mg/ml of Human Serum Albumin. Lymphocytes were enriched by Percoll gradient. For vaginal and rectal biopsies the DTT and Percoll steps were omitted and the cell suspension was passed through a 40μm nylon cell strainer. LNs were cut in small pieces and passed directly through a 40μm cell strainer. Cell suspensions were stained with the viability dye LIVE/DEAD Aqua dye (Molecular probes) before being incubated with a combination of anti-: CD4-PE Texas Red, CD3-V450, CD69-AF700, CD25-APC, CD95-AF488, CCR5- PCP-Cy5.5 (all BD Bioscience) α_4_β_7_-PeCy7 (Act-1 mAb, non-human primate repository, Beth Israel Medical Center, Boston, MA), and activated LFA-1-PE (clone MEM148, SIGMA). The LDV-FITC peptide (Tocris Bioscience) was used at a final concentration of 100nM and mixed with the antibodies. Cells were incubated with the antibodies and LDV-FITC peptide for 20 mins at 4°C, washed and fixed in 2% PFA. Greater than 200,000 events were acquired in the lymphocyte live cells gate using the BD LSRII Flow Cytometer. Data were analyzed with FlowJo 9.8.5.

### SIV viral loads

Macaque infection was confirmed by SIVgag nested PCR on PBMC as described [49]. Plasma samples were obtained from EDTA-treated whole blood and used for the determination of plasma VL by SIVgag qRT-PCR [50] (quantitative Molecular Diagnostics Core, AIDS and Cancer Virus Program Frederick National Laboratory). Tissue viral DNA and RNA loads were measured, respectively, by qPCR and qRT-PCR with standard curve method and normalized on Albumin copy number (for cell-associated viral DNA) and total RNA quantity. DNA and RNA were extracted from snap frozen tissues using DNeasy/RNeasy blood and tissue kits (Qiagen) following the manufacturer’s instructions. Primers: SIVgag FW (5’-GGTTGCACCCCCTATGACAT-3’), SIVgag RV (5’-TGCATAGCCGCTTGATGGT-3’); macaque Albumin FW (5’-ATTTTCAGCTTCGCGTCTTTTG-3’) and RV (5’-TTCTCGCTTACTGGCGTTTTCT-3’). For the DNA loads the Mastermix was ABsolute Blue Q-PCR SYBRGreen low-ROX (Thermo Fisher Scientific, Waltham, MA), while for RNA loads we used the One-step RT-qPCR Kit (KAPA Biosystems, Wilmington, MA) [14]. The PCR was run on ViiA-7 Real-Time PCR System (Thermo Fisher).

### Soluble factors

Soluble factors in plasma and in clarified vaginal and rectal swabs from uninfected and SHIV infected macaques were measured using the monkey Novex multiplex Luminex assay (Cytokine Monkey Magnetic 29-Plex Panel; Invitrogen) on a Luminex 200 instrument (Luminex Corporation, Austin, TX). Complete list of factors measured: IL1RA, CXCL11, MIF, FGF-Basic, CCL2, G-CSF, IFNγ, CCL22, IL15, CXCL8, EGF, HGF, VEGF, CXCL9, CCL5, CCL11, CCL4, CXCL10, GM-CSF, TNFα, IL1β, IL2, IL4, IL5, IL6, IL10, IL12, CCL3, IL17.

### ELN and Act-1 in vitro assays

For all experiments described in this section either RPMI 8866 cells (kindly donated by James Arthos) or primary CD4^+^ T cells or Hut78 cells (NIH AIDS Reagent Program, Division of AIDS, NIAID, NIH from Dr. Robert Gallo [51]). CD4^+^ T cells were isolated by Ficoll from buffy coat (NY blood center) followed by negative selection beads procedure (Miltenyi). After isolation, CD4^+^ T cells were activated by OKT3 (100ng/ml, eBiosciences) /IL-2 (50U/m, NCI-Frederick) and cultured in 100nM retinoic acid (RA, Sigma) to induce increased expression of α_4_β_7_. Cells were fed with IL-2 and RA every other day and used between 7 to 10 days of culture. α_4_β_7_ (Act-1, NHP-repository) or β_7_ (FIB504, ebiosciences) FACS staining were regularly performed to confirm the increase level of expression of α_4_β_7_.

To demonstrate ELN’s ability to block gp120 binding to α_4_β_7_ we used a biotinylated recombinant gp120 from HIV_SF162_ (gift from James Arthos). 100,000 RA-cultured CD4^+^ T cells were stained with the LIVE/DEAD Aqua dye (Invitrogen) and incubated or not with 10 fold increased nM concentrations of ELN for 30 mins at RT in Mn^2+^/Ca^2+^ buffer (1mM MnCl_2_, 100μM CaCl_2_, HBS: 10 mM Hepes, 150 mM NaCl_2_ in ddH_2_O). The anti-CD4 (Leu3A, BD Bioscience) was used at 2.5μg/ml to inhibit gp120 binding to CD4. Cells were then incubated with 4 μg of biotin-labeled gp120 20 min at RT in Mn^2+^/Ca^2+^ buffer, washed and then incubated with neutravidin-PE (Invitrogen, Molecular Probes) 15 mins at 4°C, washed and fixed in 2% PFA. The % of gp120 binding was measured by flow cytometry detection of PE fluorescence. All data were collected on a BD LSRII (BD Biosciences, San Jose, CA). To measure the ability of ELN to block MAdCAM-Fc (R&D Systems) was labeled with biotin using EZ-Link NHS-LC-Biotin (Thermo Scientific) kit according to manufacturer’s procedure. 100,000 LIVE/DEAD Aqua-stained RA-cultured CD4^+^ T cells were incubated or not with 4 to 5 fold increased nM concentrations of ELN in Mn^2+^/Ca^2+^ buffer for 30 mins at RT. After cells were washed, Biotin-MAdCAM-Fc (0.1 μg/well) was added for 20 mins at RT followed by neutravidin-PE for an additional 20 mins. Cells were washed, fixed in 2% PFA and the % of binding was measured by flow cytometry detection of PE fluorescence.

For the 2G3 epitope induction assays either 100,000 RPMI 8866 or RA-cultured CD4^+^ T cells were plated in Mg^2+^/Ca^2+^ buffer (1mM CaCl_2_, 1mM MgCl_2_ in HBS 0.3% BSA) and incubated with ELN or Act-1 at indicated concentrations for 15 mins at RT. PE-conjugated 2G3 Ab (gift from James Arthos; 2μg/ml final concentration) with or without Mn^2+^ (1mM final concentration) was then added 30 mins at RT. 2G3 was directly conjugated using Innova Bioscience Lightning-Link kit. Cells were washed, fixed in 1% PFA and PE fluorescence was assessed by flow cytometer. Alternatively, RA-cultured CD4^+^ T cells were first stained with CD95-V450 and CD62L-APC-Cy7 Abs for 20 mins at 4°C prior washing.

To assess 2G3 and Act-1 distinct binding site (Fig 7D), 100,000 RPMI 8866 in Ca^2+^/Mg^2+^ were incubated with increasing concentrations of unlabeled 2G3 mAb + ELN at 100nM. Cells were then stained with PE-conjugated Act-1 mAb (1μg/well), washed and fixed in 1% PFA. The mean of fluorescence of PE was measured on a BD LSRII.

To measure the ability of ELN and Act-1 to dissociate pre-bound MAdCAM-1 (Fig 8A), 100,000 RA-cultured CD4^+^ T cells were stained with LIVE/DEAD Aqua dye and resuspended in Mn^2+^/Ca^2+^ buffer. Biotinylated MadCam-1 (0.1μg) or PBS control was added for 40 mins at RT. Cells were washed once in cold assay buffer and various concentrations of unconjugated Act-1 or IgG1 or ELN + neutravidin-PE were added for 30 mins at RT. After two washes, cells were fixed in 1% PFA and acquired on a BD LSRII. The mean of Fluorescence of PE was measured on all live CD4^+^ T cells.

For the MAdCAM-1/Act-1 competition assay (Fig 8B) 100,000 RA-cultured CD4^+^ T cells were stained with LIVE/DEAD Aqua dye and resuspended in Mn^2+^/Ca^2+^ buffer. Various concentrations of recombinant MadCam-Fc-biotin were added at the same time with various concentrations of unlabeled Act-1 for 40 mins at RT. In the last 15’, neutravidin-PE was added. After two washes, cells were fixed in 1% PFA and cells were acquired on a BDLSRII. The mean of Fluorescence of PE was measured on all live CD4^+^ T cells.

To measure the effect of ELN on the levels of phospho-Y397-FAK, 1x10^6^ Hut78 (cutaneous T cell) or 8x10^6^ RA-treated CD4+ T cells were incubated for 30 mins at 37°C on fibronectin (10μg/ml) coated plate to induce detectable level of phospho-Y397-FAK. Then the cells were treated with ELN (1μM), Act-1 (500nM = 80μg /ml), IgG1 (500nM, NHP repository) or mock treated with an amount of DMSO similar to that needed to be added for ELN (10mM stock in DMSO) for 15 mins at 37°C. Cells were collected and lysed in Cell lytic M lysis reagent (Sigma) in presence of 10mM of sodium orthovanadate. Proteins were denatured in hot reducing sample buffer, separated by SDS-PAGE, transferred to a PVDF membrane and probed with a mouse anti-human phospho-Y397-FAK (clone 18/FAK, BD biosciences, for HUT78 and clone D20B1, Cell Signaling Technology for CD4+ T cells) followed by an HRP-conjugated secondary Ab. The membranes were then stripped and re-probed with a rabbit anti-human total FAK (clone D2RE2, Cell Signaling Technology) or a rabbit anti-human actin (Abcam). Protein bands were visualized using chemiluminescence detection on an Amersham 600 imager (GE Life Sciences). Relative protein expression was determined for pFAK and FAK using ImageJ software (NIH) and data was normalized against their respective control condition set as 1.

### Statistics

Unpaired non-parametric Mann-Whitney test was used to compare variables between groups of animals (CTRL, ORAL and OR+V) when the group of interests where defined before collecting the data. Kruskal-Wallis H test was used to compare groups for the Lumiex data, where the uninfected/untreated group was added after the study. Mann-Whitney was still used as post-hoc analysis of Kruskal-Wallis to avoid the stringent multiple-comparisons correction of the Dunn’s test. The paired-sample non-parametric Wilcoxon signed-rank test was used to determine significant differences during the kinetics in PK study and between conditions in the in vitro experiments. A two-tailed p = 0.05 was considered significant. The analysis was performed using Prism5a (GraphPad Software, Inc).

## Supporting Information

S1 Fig ELN has mixed effects on SHIV and HIV infection in vitro. Macaque (left) and human (right) CD4^+^ T cells were activated with okt3/IL2 and RA and treated in RA for 5–7 days. They were infected with 10 TCID50 of SHIVsf162P3 (left) or 10 TCID50 of HIVsf162 (right) per well (200,000 cells) in presence *vs* absence of different concentration of ELN (added every other day). The amount of p27 (left) and p24 (right) in culture supernatant was measured by ELISA (ZeptoMetrix Corp.). One representative experiment out of 3 is shown.
(TIF)

S2 Fig α_4_β_7_ gating strategy and ELN/LDV-FITC competition curve. A) Gating strategy of α_4_β_7_^high^ CD4 T cells in PBMC. Mononuclear cells were gated on live and CD3^+^ CD4^+^ cells. CD95 was used to help with the identification of the α_4_β_7_^high^, positive and negative populations. B) Typical standard curve used in parallel with each receptor occupancy measurement in the pharmacodynamics studies. PBMCs (300,000/well) were incubated 20’ at 4°C with different amounts of ELN in Mg^2+^/Ca^2+^ buffer and washed twice before staining with the LDV-FITC and antibody mix. The standard curve was prepared and stained in parallel with PBMCs or mononuclear cells isolated from tissues of monkeys treated with ELN before and after treatment: cells were stained with the LIVE/DEAD dye Aqua, washed and incubated with the antibody mix including 100nM of LDV-FITC or a non-specific LLA tripeptide (only for the highest standard) in Mg^2+^/Ca^2+^ buffer. Gating was done on singlets, live, CD3^+^ CD4^+^. MFI of LDV-FITC was plotted. C) Representative plots showing the frequencies of α_4_β_7_^high^ cells within live, CD3^+^ CD4^+^ cells from vaginal and rectal tissue of 1 animal before and 48hrs after treatment with 20mg/Kg of ELN orally.
(TIF)

S3 Fig Orally administered ELN (by oral gavage and capsules) decreases inflammatory CC/CK in blood, vaginal and rectal fluids. A) ELN was given by oral gavage to 8 animals. All animals were sampled at baseline (BL), 4 were sampled at 24h and 4 at 48h. A) The concentrations of soluble factors modulated by ELN treatment are shown in rectal (top panel) and vaginal (bottom panel) swabs. Significance (Wilcoxon t-test two-tails α<0.05) of pre-post-treatment comparison is reached when the 24h and 48h are pooled. B-C) 3 macaques were administered 1 capsule of 100mg of ELN for 2 consecutive days and concentrations of soluble factors were measured 24hr after the 2^nd^ administration (48h after the 1^st^ capsule). The concentration of soluble factors that appeared to be modulated by ELN in blood (B) and all those that were detectable in rectal and vaginal fluids are shown.
(TIF)

S4 Fig CC/CK measurement in plasma at necropsy. Concentration of soluble factors in plasma at necropsy for infected and uninfected (n = 6) animals. Specifically, here are shown all the soluble factors that were found significantly modulated by SHIV infection at 3 weeks p.i. (Fig 5A) and those that were found to differ significantly by Kruskal-Wallis in one of the groups. Mann-Whitney p values are shown only for the factors found to differ significantly by Kruskal-Wallis.
(TIF)

S5 Fig ELN tends to increase depletion of CCR5^+^ CD4^+^ T cells in tissues. The frequency of CCR5^+^ cells within live, singlets CD3^+^ CD4^+^ T cells are shown for different tissue at the time of necropsy of the SHIV infected animals. Bars represent mean ± SEM. p< 0.05 is considered significant. p<0.125 are shown to indicate tendency toward significance.
(TIF)

S6 Fig ELN activates, while Act-1 inhibits α_4_β_7_ integrin *in vitro*. RPMI8866 cells plated in Ca^2+^/Mg^2+^ assay buffer were incubated with indicated concentrations of ELN (A) or Act-1 mAb (B). Cells were then stαined with PE-conjugated 2G3 mAb ± Mn^2+^ (1mM). Geometric MFI (GMFI) of 2G3-PE was plotted.
(TIF)

S7 Fig 2G3 and Act-1 bind to distinct α_4_β_7_ site. RPMI 8866 in Ca^2+^/Mg^2+^ were incubated with increasing concentrations of unlabeled 2G3 mAb. Cells were then stained with PE-conjugated Act-1 mAb (1μg/well). Geometric MFI (GMFI) of Act-1-PE was plotted.
(TIF)

S8 Fig Act-1 does not completely block MAdCAM-1 interaction with α_4_β_7_. LIVE/DEAD Aqua stained RA-cultured CD4^+^ T cells or RPMI8866, in Mn^2+^/Ca^2+^ buffer, were stained or not with a saturating amount (1μg) of APC-conjugated Act-1 mAb. After one wash, indicated concentrations of MadCam-Fc-biotin (above each plot) were added for 40 mins at RT. Cells were washed and neutravidin-PE was added for 10 mins on ice. Frequency of single and double positive (APC+ PE+) cell population are indicated in each quadrant. MFI of APC and PE in each condition remain constant. Results are representative of two buffy coat donors for CD4+ T cells and duplicates for RPMI8866. A reduction in MAdCAM-1 signal with concentrations above 100ng/well were consistent in all the experiment.
(TIF)

S9 Fig ELN increases FAK phosphorylation. RA-treated Hut78 cells were incubated with ELN (1μM), Act-1 (500nM), IgG1 (500nM) or control (mock treated with DMSO) for 15 mins at 37°C on fibronectin coated wells. Cells were lysed and phospho-Y397-FAK, FAK and β actin expression were determined by western blotting. Relative protein expression was determined for pFAK and FAK using ImageJ. The data were normalized against FAK expression. Results from 3 independent experiments done with Hut-78 cells were expressed as fold change in pY397 FAK after normalization to their respective control (set as 1). Mean ± SEMs are shown. p< 0.05 is considered significant.
(TIF)

S10 Fig ELN or Act-1 does not modulate the phenotype of CD4^+^ T cells in vitro. PBMCs (A) or isolated CD4^+^ T cells (B) from 6 buffy coat donors were activated with coated OKT3 and treated for 24h with ELN (1μM), Act-1 (500nM), IgG1 (500nM) or mock treated with DMSO (CTRL). The cells were then stained with a LIVE/DEAD Aqua and an antibody combination of anti-: CD4, CD3, CD69, CD25, CD95, CCR5 and activated LFA-1 (clone MEM148). The expression of indicated marker within live, singlets, CD3^+^ CD4^+^ T cells are shown. Results were expressed as fold change MFI or % after normalization to their respective control (set as 1). CCR5 and activated LFA-1 markers were below specific detectable level in these samples.
(TIF)
