# Correction: A Small Molecule, Which Competes with MAdCAM-1, Activates Integrin α_4_β_7_ and Fails to Prevent Mucosal Transmission of SHIV-SF162P3

**DOI:** 10.1371/journal.ppat.1005781

**Published:** 2016-07-14

**Authors:** Géraldine Arrode-Brusés, Diana Goode, Kyle Kleinbeck, Jolanta Wilk, Ines Frank, Siddappa Byrareddy, James Arthos, Brooke Grasperge, James Blanchard, Thomas Zydowsky, Agegnehu Gettie, Elena Martinelli

In the Author Contributions section, Géraldine Arrode-Brusés (GAB) should be listed as one of the persons who wrote the paper.
