# Supplementary figures and images for: A Small Molecule, Which Competes with MAdCAM-1, Activates Integrin α4β7 and Fails to Prevent Mucosal Transmission of SHIV-SF162P3
Source: PLoS Pathog. 2016 Jun 27;12(6):e1005720. doi: 10.1371/journal.ppat.1005720 (PMC4922556; doi:10.1371/journal.ppat.1005720)

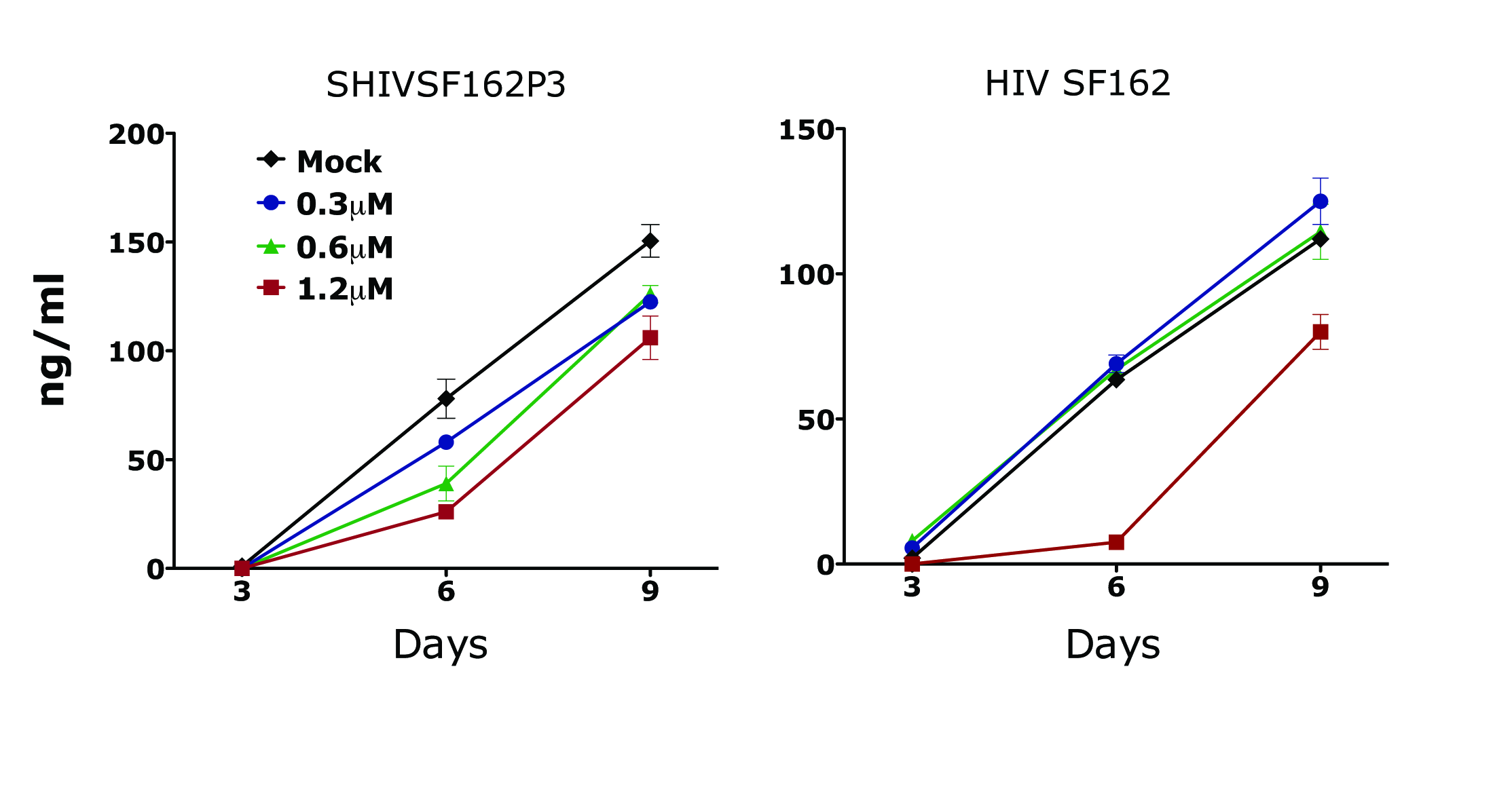

Supplement: S1 Fig — Macaque (left) and human (right) CD4+ T cells were activated with okt3/IL2 and RA and treated in RA for 5–7 days. They were infected with 10 TCID50 of SHIVsf162P3 (left) or 10 TCID50 of HIVsf162 (right) per well (200,000 cells) in presence vs absence of different concentration of ELN (added every other day). The amount of p27 (left) and p24 (right) in culture supernatant was measured by ELISA (ZeptoMetrix Corp.). One representative experiment out of 3 is shown. (TIF) [file ppat.1005720.s001.tif]

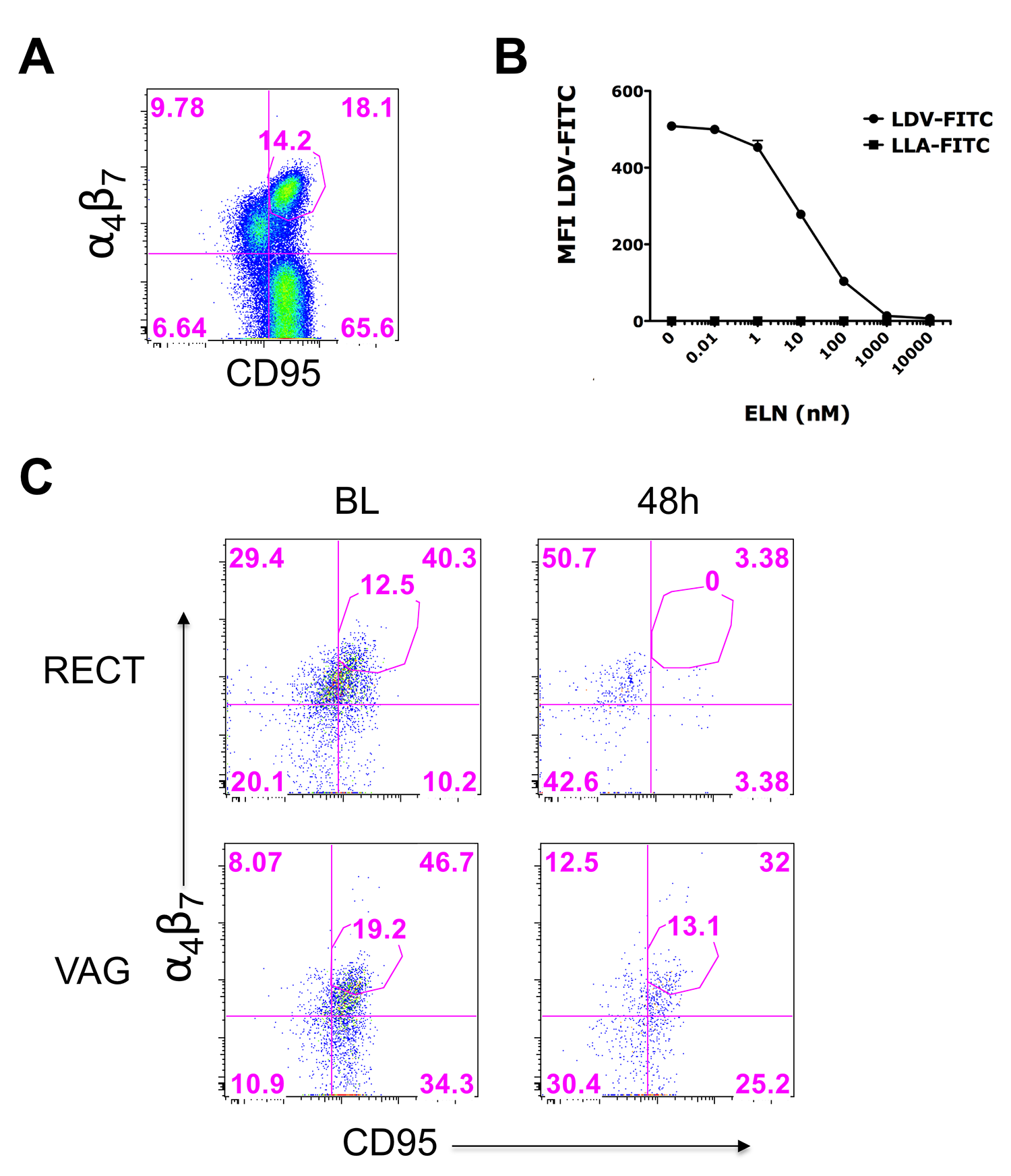

Supplement: S2 Fig — A) Gating strategy of α4β7 high CD4 T cells in PBMC. Mononuclear cells were gated on live and CD3+ CD4+ cells. CD95 was used to help with the identification of the α4β7 high, positive and negative populations. B) Typical standard curve used in parallel with each receptor occupancy measurement in the pharmacodynamics studies. PBMCs (300,000/well) were incubated 20’ at 4°C with different amounts of ELN in Mg2+/Ca2+ buffer and washed twice before staining with the LDV-FITC and antibody mix. The standard curve was prepared and stained in parallel with PBMCs or mononuclear cells isolated from tissues of monkeys treated with ELN before and after treatment: cells were stained with the LIVE/DEAD dye Aqua, washed and incubated with the antibody mix including 100nM of LDV-FITC or a non-specific LLA tripeptide (only for the highest standard) in Mg2+/Ca2+ buffer. Gating was done on singlets, live, CD3+ CD4+. MFI of LDV-FITC was plotted. C) Representative plots showing the frequencies of α4β7 high cells within live, CD3+ CD4+ cells from vaginal and rectal tissue of 1 animal before and 48hrs after treatment with 20mg/Kg of ELN orally. (TIF) [file ppat.1005720.s002.tif]

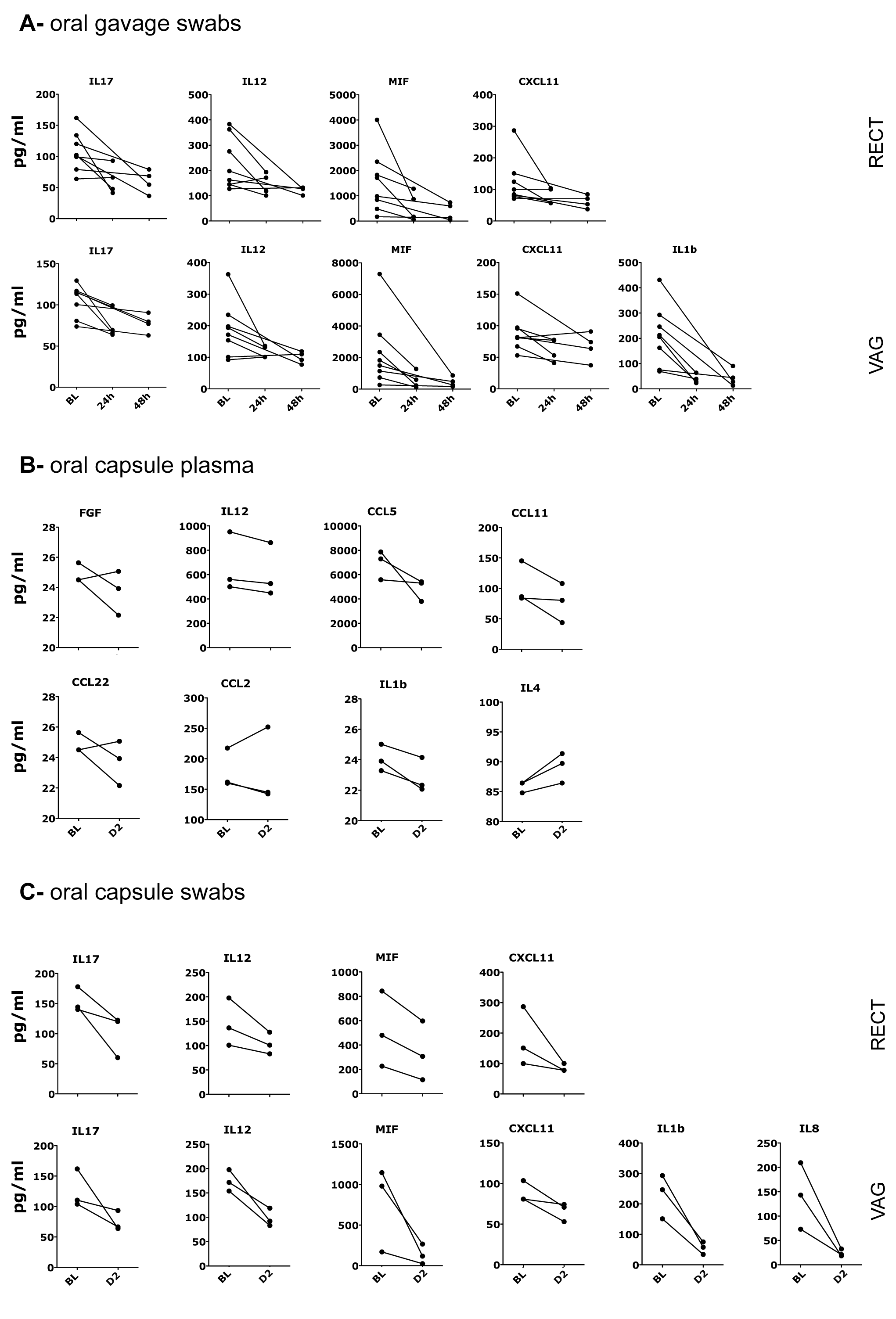

Supplement: S3 Fig — A) ELN was given by oral gavage to 8 animals. All animals were sampled at baseline (BL), 4 were sampled at 24h and 4 at 48h. A) The concentrations of soluble factors modulated by ELN treatment are shown in rectal (top panel) and vaginal (bottom panel) swabs. Significance (Wilcoxon t-test two-tails α<0.05) of pre-post-treatment comparison is reached when the 24h and 48h are pooled. B-C) 3 macaques were administered 1 capsule of 100mg of ELN for 2 consecutive days and concentrations of soluble factors were measured 24hr after the 2nd administration (48h after the 1st capsule). The concentration of soluble factors that appeared to be modulated by ELN in blood (B) and all those that were detectable in rectal and vaginal fluids are shown. (TIF) [file ppat.1005720.s003.tif]

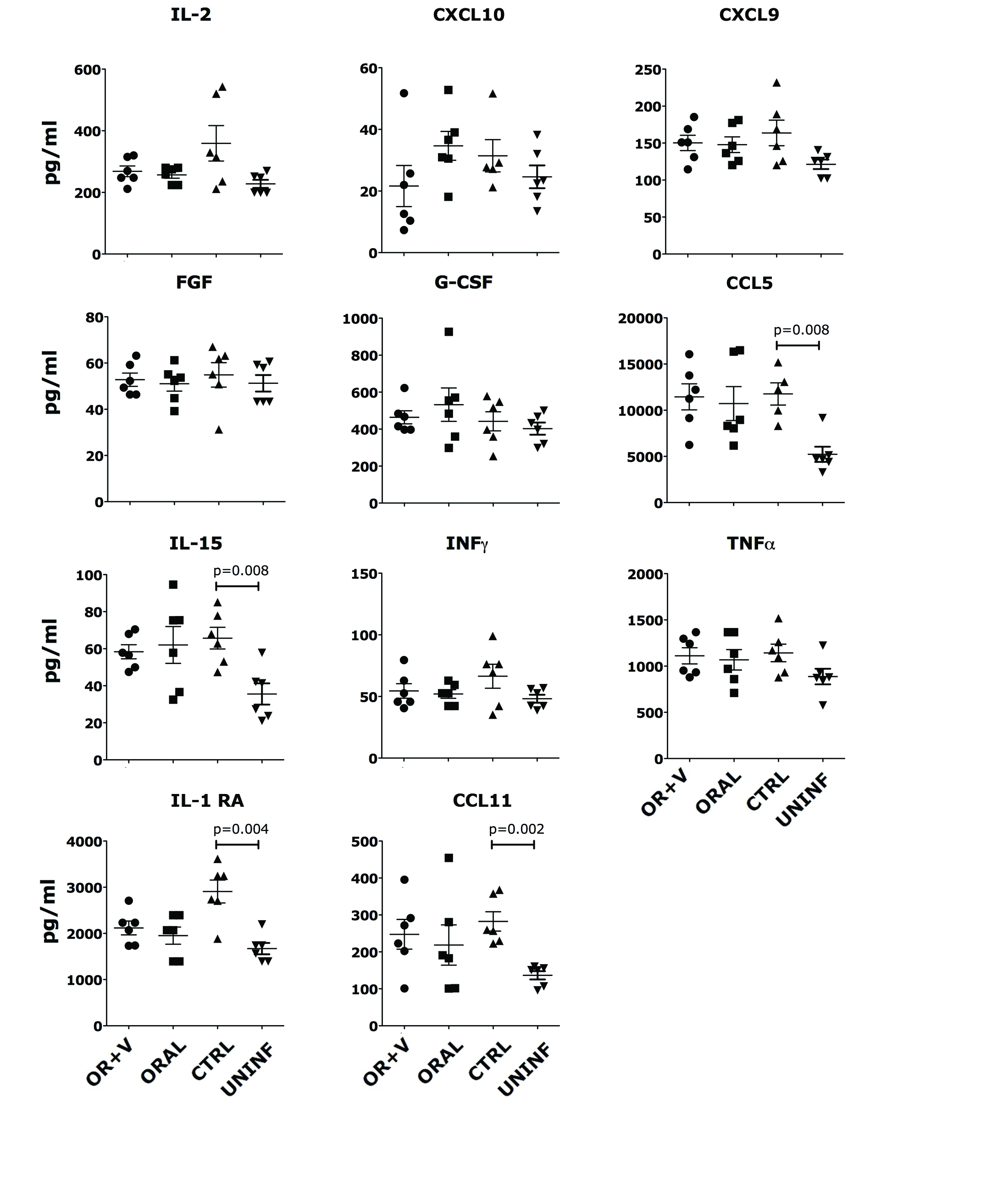

Supplement: S4 Fig — Concentration of soluble factors in plasma at necropsy for infected and uninfected (n = 6) animals. Specifically, here are shown all the soluble factors that were found significantly modulated by SHIV infection at 3 weeks p.i. (Fig 5A) and those that were found to differ significantly by Kruskal-Wallis in one of the groups. Mann-Whitney p values are shown only for the factors found to differ significantly by Kruskal-Wallis. (TIF) [file ppat.1005720.s004.tif]

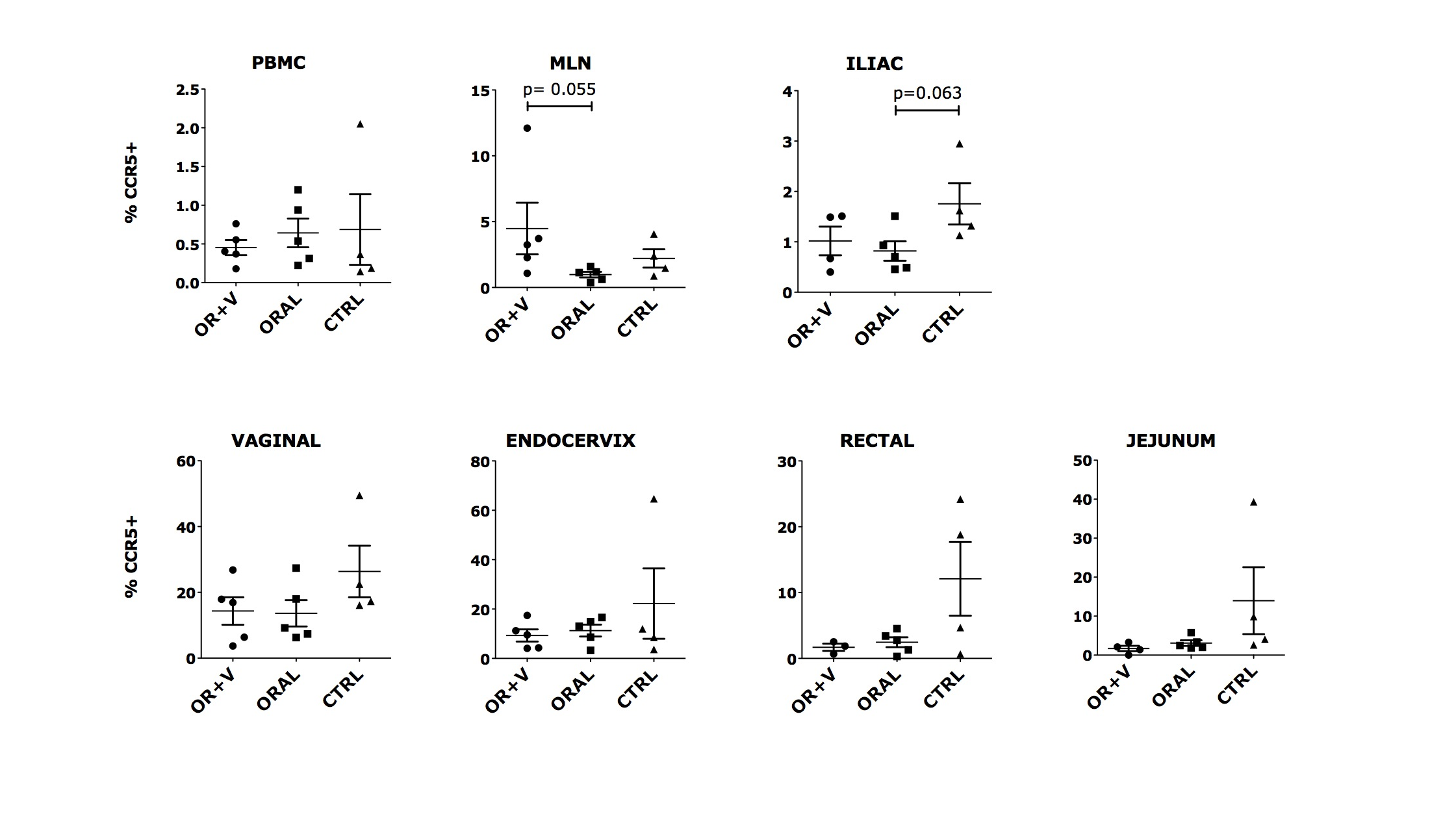

Supplement: S5 Fig — The frequency of CCR5+ cells within live, singlets CD3+ CD4+ T cells are shown for different tissue at the time of necropsy of the SHIV infected animals. Bars represent mean ± SEM. p< 0.05 is considered significant. p<0.125 are shown to indicate tendency toward significance. (TIF) [file ppat.1005720.s005.tif]

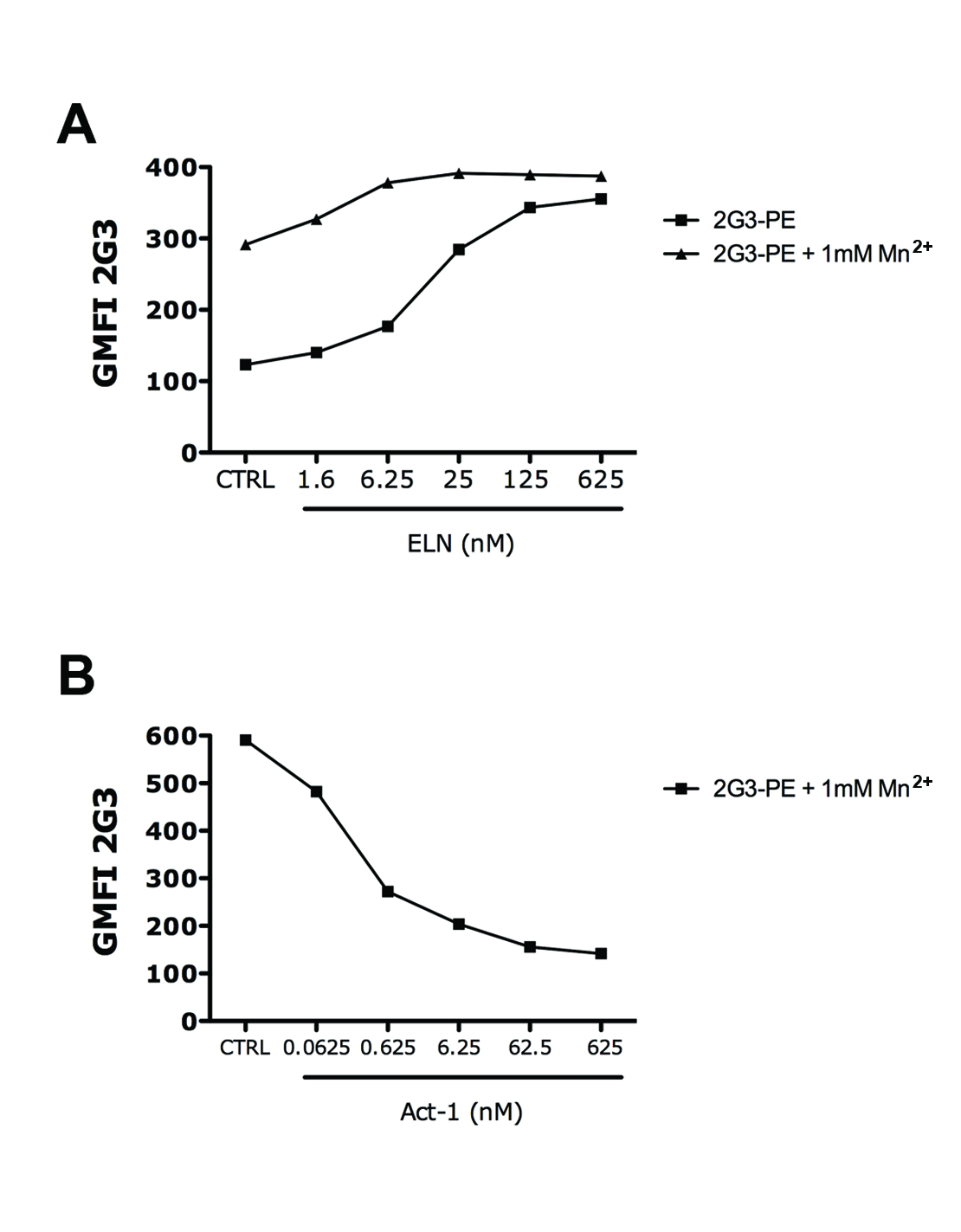

Supplement: S6 Fig — RPMI8866 cells plated in Ca2+/Mg2+ assay buffer were incubated with indicated concentrations of ELN (A) or Act-1 mAb (B). Cells were then stαined with PE-conjugated 2G3 mAb ± Mn2+ (1mM). Geometric MFI (GMFI) of 2G3-PE was plotted. (TIF) [file ppat.1005720.s006.tif]

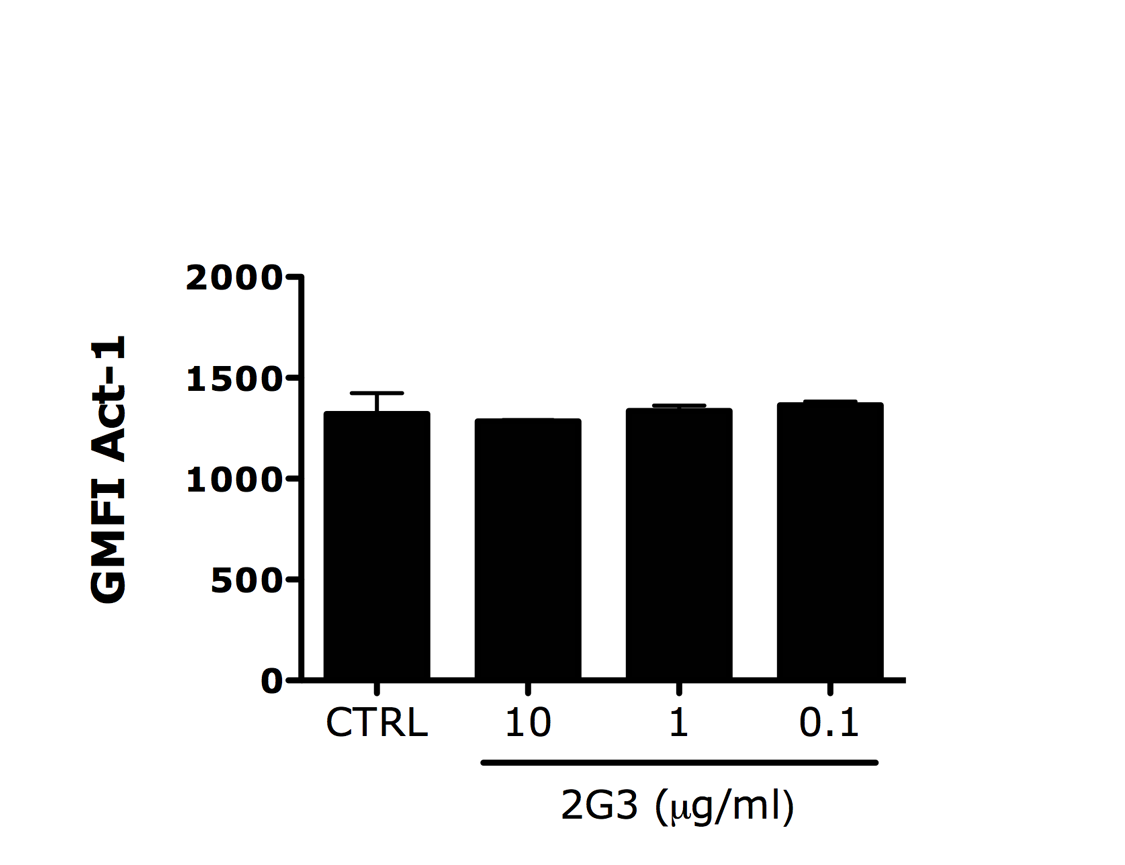

Supplement: S7 Fig — RPMI 8866 in Ca2+/Mg2+ were incubated with increasing concentrations of unlabeled 2G3 mAb. Cells were then stained with PE-conjugated Act-1 mAb (1μg/well). Geometric MFI (GMFI) of Act-1-PE was plotted. (TIF) [file ppat.1005720.s007.tif]

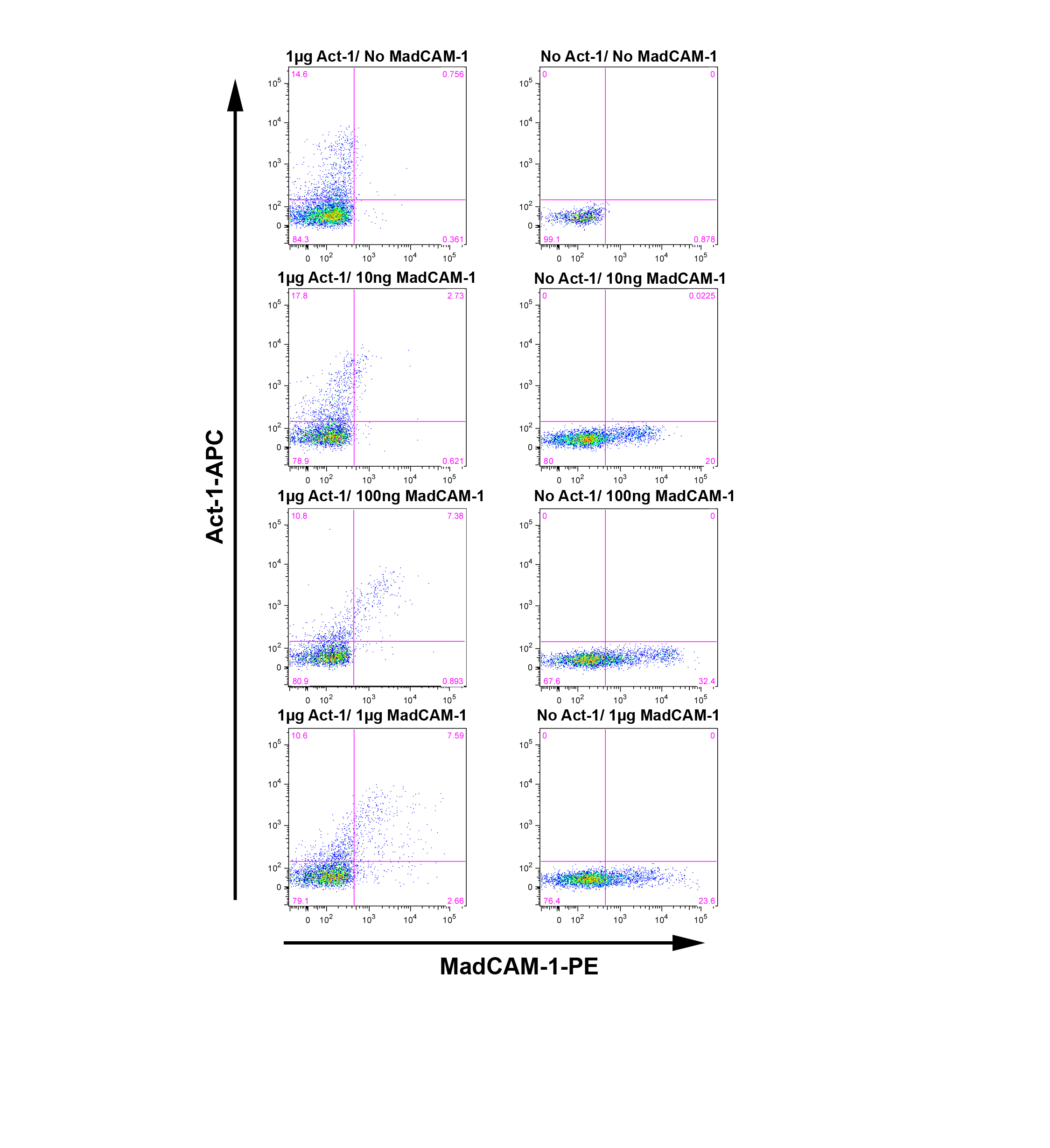

Supplement: S8 Fig — LIVE/DEAD Aqua stained RA-cultured CD4+ T cells or RPMI8866, in Mn2+/Ca2+ buffer, were stained or not with a saturating amount (1μg) of APC-conjugated Act-1 mAb. After one wash, indicated concentrations of MadCam-Fc-biotin (above each plot) were added for 40 mins at RT. Cells were washed and neutravidin-PE was added for 10 mins on ice. Frequency of single and double positive (APC+ PE+) cell population are indicated in each quadrant. MFI of APC and PE in each condition remain constant. Results are representative of two buffy coat donors for CD4+ T cells and duplicates for RPMI8866. A reduction in MAdCAM-1 signal with concentrations above 100ng/well were consistent in all the experiment. (TIF) [file ppat.1005720.s008.tif]

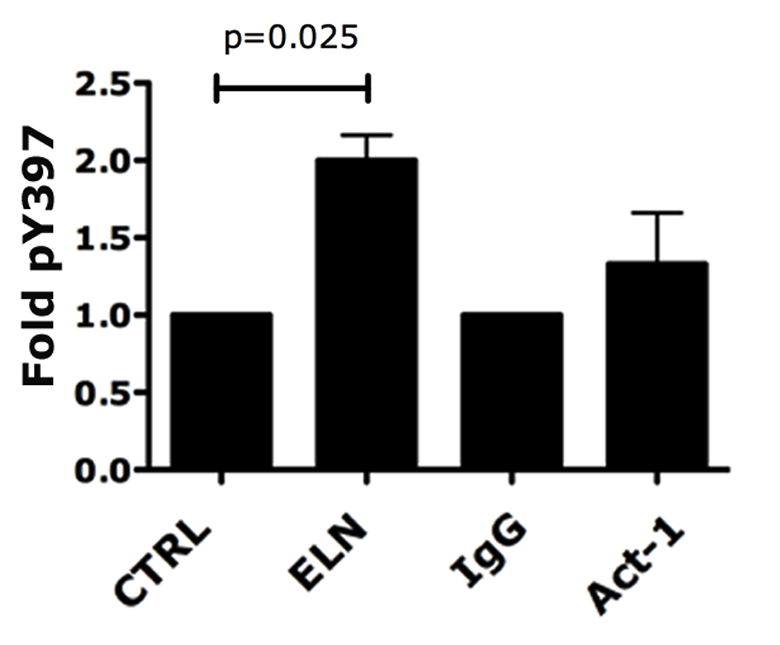

Supplement: S9 Fig — RA-treated Hut78 cells were incubated with ELN (1μM), Act-1 (500nM), IgG1 (500nM) or control (mock treated with DMSO) for 15 mins at 37°C on fibronectin coated wells. Cells were lysed and phospho-Y397-FAK, FAK and β actin expression were determined by western blotting. Relative protein expression was determined for pFAK and FAK using ImageJ. The data were normalized against FAK expression. Results from 3 independent experiments done with Hut-78 cells were expressed as fold change in pY397 FAK after normalization to their respective control (set as 1). Mean ± SEMs are shown. p< 0.05 is considered significant. (TIF) [file ppat.1005720.s009.tif]

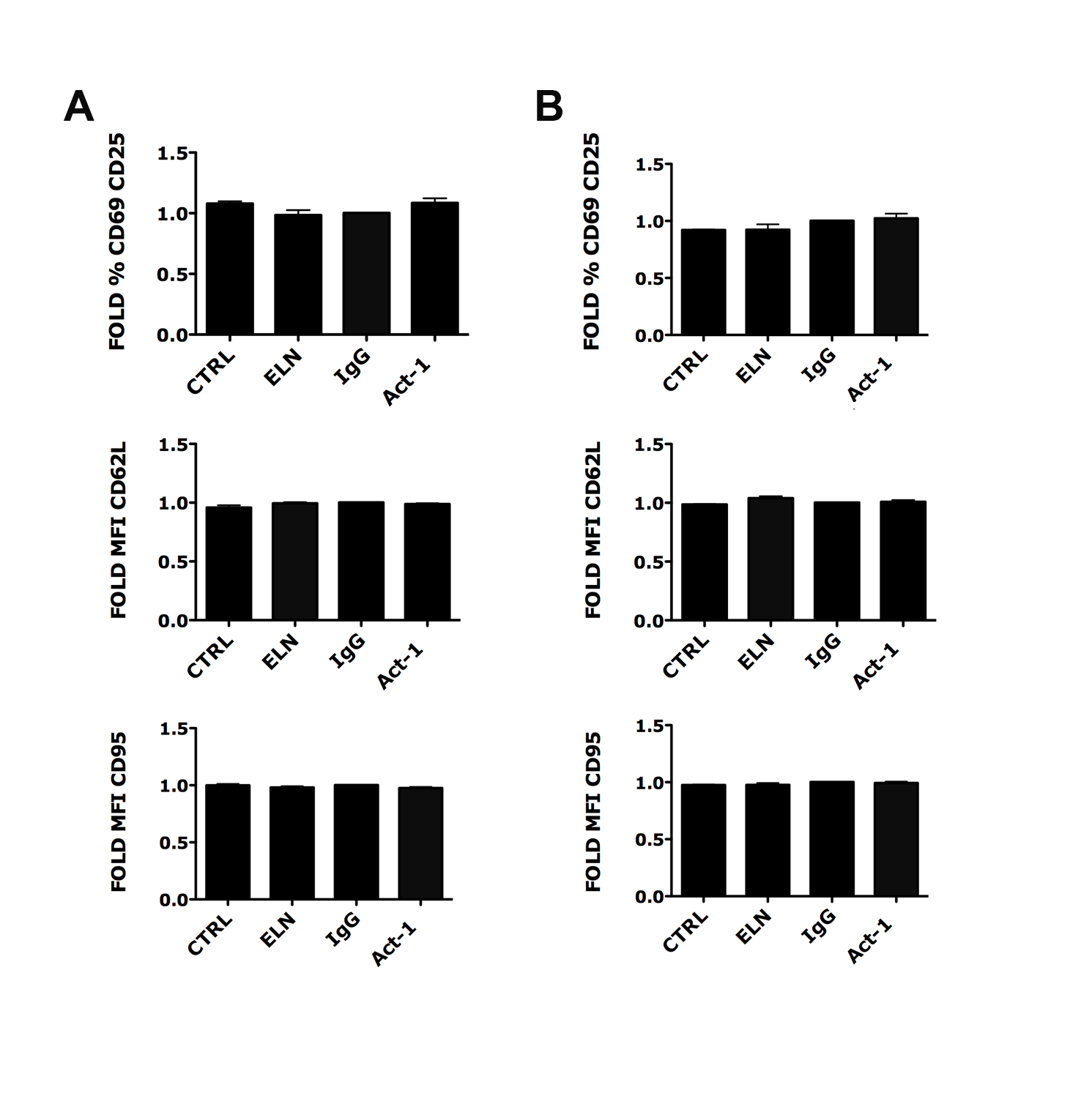

Supplement: S10 Fig — PBMCs (A) or isolated CD4+ T cells (B) from 6 buffy coat donors were activated with coated OKT3 and treated for 24h with ELN (1μM), Act-1 (500nM), IgG1 (500nM) or mock treated with DMSO (CTRL). The cells were then stained with a LIVE/DEAD Aqua and an antibody combination of anti-: CD4, CD3, CD69, CD25, CD95, CCR5 and activated LFA-1 (clone MEM148). The expression of indicated marker within live, singlets, CD3+ CD4+ T cells are shown. Results were expressed as fold change MFI or % after normalization to their respective control (set as 1). CCR5 and activated LFA-1 markers were below specific detectable level in these samples. (TIF) [file ppat.1005720.s010.tif]
